# Atomic cerium modulated palladium nanoclusters exsolved ferrite catalysts for lean methane conversion

**DOI:** 10.1002/EXP.20220060

**Published:** 2022-07-11

**Authors:** Yanling Yang, Si Wang, Xin Tu, Zhiwei Hu, Yinlong Zhu, Hongquan Guo, Zhishan Li, Li Zhang, Meilan Peng, Lichao Jia, Meiting Yang, Guangming Yang, Xurong Qiao, Jiahui Sun, Xiaolu Liang, Zhen Zhang, Yanru Zhu, Lei Shi, Chenxing Jiang, Yingru Zhao, Jianhui Li, Zongping Shao, Xin Zhang, Yifei Sun

**Affiliations:** ^1^ College of Energy Xiamen University Xiamen China; ^2^ Beijing State Key Laboratory of Chemical Resource Engineering Beijing Advanced Innovation Center for Soft Matter Science and Engineering Beijing University of Chemical Technology Beijing China; ^3^ Department of Electrical Engineering and Electronics University of Liverpool Liverpool UK; ^4^ Max Planck Institute for Chemical Physics of Solids Dresden Germany; ^5^ Institute for Frontier Science Nanjing University of Aeronautics and Astronautics Nanjing China; ^6^ School of Materials Science and Engineering, State Key Lab of Material Processing and Die & Mould Technology Huazhong University of Science and Technology Wuhan China; ^7^ State Key Laboratory of Materials‐Oriented Chemical Engineering, College of Chemical Engineering Nanjing Tech University Nanjing China; ^8^ State Key Laboratory for Mechanical Behavior of Materials Xi'an Jiaotong University Xi'an China; ^9^ School of Chemical Engineering Dalian University of Technology Dalian China; ^10^ National Engineering Laboratory for Green Chemical Productions of Alcohols‐Ethers‐Esters, College of Chemistry and Chemical Engineering Xiamen University Xiamen China; ^11^ State Key Laboratory of Physical Chemistry of Solid Surface Xiamen University Xiamen China; ^12^ Shenzhen Research Institute of Xiamen University Shenzhen Guangdong China

**Keywords:** Ce incorporation, in situ exsolution, methane conversion, Pd nanocluster, perovskite

## Abstract

The active and stable palladium (Pd) based catalysts for CH_4_ conversion are of great environmental and industrial significance. Herein, we employed N_2_ as an optimal activation agent to develop a Pd nanocluster exsolved Ce‐incorporated perovskite ferrite catalyst toward lean methane oxidation. Replacing the traditional initiator of H_2_, the N_2_ was found as an effective driving force to selectively touch off the surface exsolution of Pd nanocluster from perovskite framework without deteriorating the overall material robustness. The catalyst showed an outstanding T_50_ (temperature of 50% conversion) plummeting down to 350°C, outperforming the pristine and H_2_‐activated counterparts. Further, the combined theoretical and experimental results also deciphered the crucial role that the atomically dispersed Ce ions played in both construction of active sites and CH_4_ conversion. The isolated Ce located at the A‐site of perovskite framework facilitated the thermodynamic and kinetics of the Pd exsolution process, lowering its formation temperature and promoting its quantity. Moreover, the incorporation of Ce lowered the energy barrier for cleavage of C─H bond, and was dedicated to the preservation of highly reactive PdO*_x_* moieties during stability measurement. This work successfully ventures uncharted territory of in situ exsolution to provide a new design thinking for a highly performed catalytic interface.

## Graphical abstract

The CH_4_ conversion is of great environmental and industrial significance to solve the greenhouse effect. N_2_ as an optimal activation agent to develop a Pd nanocluster exsolved Ce‐incorporated perovskite ferrite catalyst toward lean methane oxidation. The isolated Ce locating at the A‐site of perovskite framework facilitates the thermodynamic and kinetics of the Pd exsolution process, lowering the Pd nanocluster formation temperature and promoting its quantity.

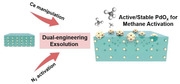

## 1 INTRODUCTION

The surging minable reserves of natural gas and shale gas have led to global interest in the use of methane for vehicles, power plants, and even fuel cells (e.g., solid oxide fuel cells (SOFCs)).^[^ ^1^ ^]^ However, the exponentially grown market of methane results in threaten of greenhouse gases, since methane is a notorious greenhouse gas with more than 20 times global warming effect than carbon dioxide. In view of the mitigating climate crisis and anthropogenic carbon footprint, it is imperative to suppress methane emissions and convert unburnt methane before it is released into nature. Direct flame combustion is currently employed in the natural gas industry and SOFC systems to manage potential methane release.^[^ ^2^ ^]^ But high temperature flaming inevitably emits toxic byproducts, such as SO*_x_* and NO*_x_*, which is harmful to public health. Therefore, to meet the target of energy conservation and emission reduction, it is urgently needed to design highly performed catalysts with low light‐off temperature and stable architecture for methane oxidation.^[^ ^3^ ^]^

Palladium (Pd) is regarded as the most favorable metal for activating the C─H bond in lean combustion conditions at low temperature (<300°C), whose compositional modality and interaction with support are vital parameters affecting catalytic performance.^[^ ^4^ ^]^ Nowadays, the consensus has been reached that a metastable structure of PdO*_x_* (or the hybrid of Pd‐PdO*_x_*) delivers higher activities for methane combustion, rather than fully oxidized (PdO_2_) or metallic Pd.^[^ ^5^ ^]^ In the traditional manufacture principle, strong electrostatic adsorption or impregnation is the comment method to deposit the active Pd species on metal oxides supports,^[^ ^6^ ^]^ while the Pd nanocluster demonstrates better activity than the single atom and nanoparticle counterparts. By varying the loading mass, Murata found that Pd particle size‐dependent turnover frequency (TOF) follows a volcano‐shaped trend.^[^ ^7^ ^]^ Fayet et al. reported that the Pd nanoclusters with up to 25 atoms demonstrated the highest activity for methane activation, as compared to other counterparts with fewer Pd atom numbers.^[^ ^8^ ^]^ This result is partially supported by the theoretical study that the energy barrier for breaking the C─H bond in methane on Pd dimer is lower than the Pd single atom.^[^ ^9^ ^]^ Fujimoto et al. reported that strong Pd─O bonds in ultra‐small PdO*_x_* (<1 nm) also lead to a decrease in the TOF of methane oxidation.^[^ ^10^ ^]^

**FIGURE 1:**
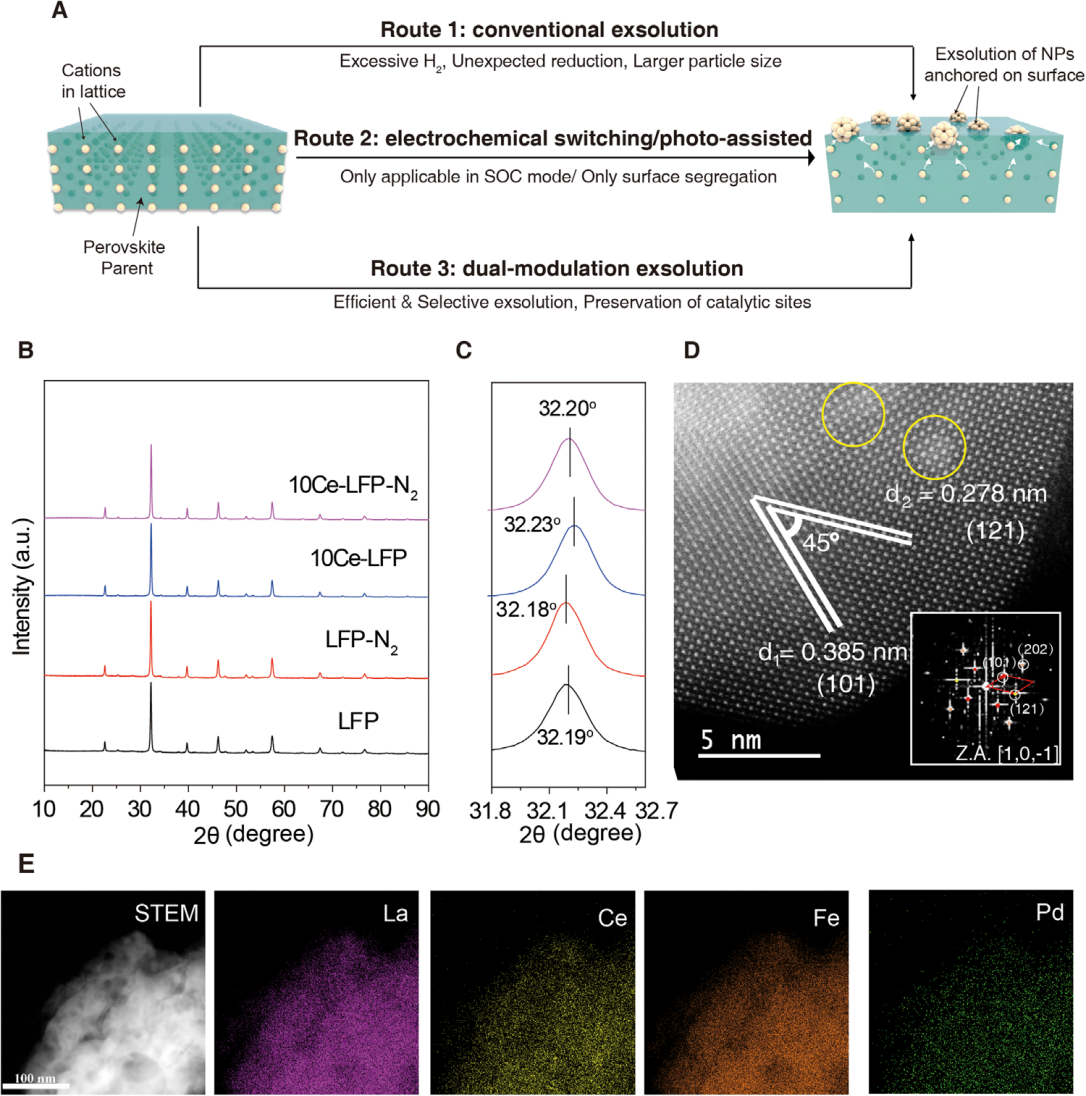
The modality of Pd nanocluster exsolved perovskite catalyst. (A) The schematic of different pathways for the realization of in situ exsolution metallic nanoparticles from perovskite. The light yellow spots in left schematic diagram indicate the cations uniformly distributed in lattice. The light green cuboid represents the perovskite parent. After in situ treatment, the exsolved nanoparticles on the surface of perovskite can be found, represented in light yellow segregated spots. (B) The powder XRD pattern of various catalysts. (C) Zoom‐in XRD plots from 31.8° to 32.7°. (D) Aberration corrected transmission electron microscopy (AC‐TEM) image with the corresponding FFT pattern of 10Ce‐LFP‐N_2_ catalyst. (E) High‐angle annular dark field scanning transmission electron microscopy (HAADF‐STEM) and corresponding energy dispersive X‐ray spectroscopy (EDX) mapping images of 10Ce‐LFP‐N_2_ catalyst

However, the poor adhesion of Pd nanocluster to support coherently leads to the unexpected reduction, deep oxidation, sintering, decomposition, and irreversible loss of reactive surface area. Goodman discovered that supported Pd nanoparticles rapidly lose activity for methane oxidation due to decomposition into inactive single atoms at high‐temperature.^[^ ^11^ ^]^ To address the challenge of stability, endeavors for conserving Pd active sites upon prolonged test have been eagerly pursued.^[^ ^12^, ^13^, ^14^, ^15^, ^16^ ^]^ Recently, plenty of studies have demonstrated a post‐growth strategy, called in situ exsolution, to construct robust metal/oxide (especially perovskite oxide) interfaces.^[^ ^17^ ^]^ Specifically, oxide lattice can evenly accommodate catalytic active cations during annealing and exsolves them to form metallic nanoparticles (NPs) in reducing environments such as H_2_, photo irradiation, and electrical field (Figure 1A, route 1 and 2).^[^ ^18^, ^19^, ^20^, ^21^ ^]^ The exsolved NPs are uniformly pinned into perovskite matrix with a partially imbedded/partially exposed modality, assuring a robust catalytic interface. In situ exsolution can alleviate the persistent problems that traditional impregnated metal/support suffers, that is, inconvenience, uneven particle distribution, and weak adhesion.^[^ ^22^, ^23^ ^]^

Herein, we reported a Pd nanocluster exsolved perovskite ferrite catalyst which delivered an impressive catalytic activity (*T*_50_ = 350°C) and stability for lean methane combustion. The N_2_ gas with intermediate oxygen partial pressure (P_O2_ of 10^–3^ Pa) was employed as an optimal activation agent, replacing the wildly used one of H_2_, for in situ exsolution, which selectively stripped off the oxygen ions and triggered the formation of Pd nanoclusters (∼2–3 nm) without destroying the catalyst integrity (Figure 1A, route 3). Meanwhile, the atomically dispersed Ce cations in the perovskite framework were found to be conducive to promoting the reduction of Pd^4+^ ions to metallic Pd^0^ thermodynamically and kinetically. During CH_4_ conversion, the existence of Ce not only promoted the activation of methane, but also served as an oxygen reservoir to accelerate the reaction kinetics via M‐K mechanism. More importantly, the Ce incorporation greatly contributed to the preservation of reactive PdO*_x_* phase with desirable stability. While its counterpart without Ce was deeply oxidized to form a large amount of less‐reactive Pd^4+^ species, resulting in quick loss of initial activity.

## 2 RESULTS AND DISCUSSION

### 2.1 Structural characterizations

A series of Ce‐doped LaFe_0.97_Pd_0.3_O_3_ perovskite catalyst were prepared by a sol‐gel method and the influence of Ce content on crystallinity of the material was preliminarily analyzed by X‐ray diffraction pattern (XRD). Plenty of previous works have reported the incorporation of Pd element into B‐site of LaFeO_3_ perovskite with the concentration as high as 5 mol%.^[^ ^17^ ^]^ Therefore, it should be reasonable to expect the full incorporation of 3 mol% Pd in our material system. In our work, all catalysts demonstrate clear diffraction peaks ascribed to orthorhombic perovskite, suggesting the formation of pure phase (Figure 1B,C and Figure S1). The main peak of (110) plane shifts from 32.19° to 32.23° upon the integration of optimal 10 mol% Ce into A‐site of perovskite (space group: Pmna, lattice parameter: *a* = 5.567 Å, *b* = 7.850 Å, *c* = 5.559 Å, shown in Figure S2), indicating the decrease of lattice volume probably due to the ionic radius difference between La^3+^ (101.6 pm) and Ce^4+^ (92.0 pm). Generally, the reducibility of cation is one of the most important parameters influencing in situ exsolution. And as the practical tool to identify this physical property, H_2_‐TPR was performed on Ce‐doped catalysts (Figure S3) and the results illustrate that adding 10 mol% Ce not only drastically shifts the reduction peak of Pd^4+^ to Pd° from ∼220°C to ∼50°C, but also drastically accelerates the reduction rate. This result preliminarily proves that the existence of Ce in lattice facilitates the reduction of Pd thermodynamically and kinetically.

At the atomic scale, the mechanism of metal exsolution from perovskite lattice involves multi‐step charge transfer and ion diffusion. Essentially, the metal exsolution is a phase separation phenomenon that is controlled and influenced by various lattice parameters and external conditions. Upon exposure to a reducing atmosphere (e.g., hydrogen) with ultra‐low oxygen partial pressure (P_O2_) (usually <10^–21^ Pa), oxygen ions are rapidly stripped from the surface of archetypal perovskite, resulting in mass loss and the formation of oxygen vacancies (Equation (1)):

(1) $$O^{2-}\to V_{O}.+2e^{-}+\frac{1}{2}O_{2}$$

As the reaction proceeds, the average oxidative valence of reducible B‐site cations decreases since they uptake the electrons released from oxygen ions to keep electron neutrality. On the other hand, the as‐formed oxygen vacancies destabilize the balance of lattice stoichiometry, which will be eventually compromised by the metal nucleation on the surface. As noted and reported before, the exsolution was typically triggered in H_2_ atmosphere at high temperatures.^[^ ^17^ ^]^ Therefore, following this traditional way, we first treated the 10Ce‐LFP catalyst with 5% H_2_/N_2_ at 800°C for 3 h. The high‐resolution TEM confirms the lattice fringe of perovskite is maintained and well defined, but the high angle annular dark field‐scanning transmission electron microscope (HAADF‐STEM) and corresponding EDX mapping results unveil the segregation of exsolved Pd nanoparticles with a large diameter of 15–20 nm which may retard the fast methane conversion (Figure S4). Indeed, the main restraint of in situ exsolution by H_2_ includes two points: (1) the reaction of H_2_ and as‐formed O_2_ from stripped oxygen ions will accelerate the destabilization of perovskite lattice, accelerating the cation nucleation and overgrowth of the nanoparticles. (2) Such a critical atmosphere usually results in the unwanted decomposition of oxide (e.g., reduction of Fe^3+^ to Fe^2+^ or Fe^0[^ ^24^ ^]^) and irreversible deactivation of the catalyst.

To migrate the obstacle, herein, the atmospheres with relatively higher P_O2_ were employed as the alternative and mild driving force providing more control over exsolution. The P_O2_ values of different atmospheres were measured by a homemade solid‐state oxygen gradient cell (Figure S5). Using air in the counter electrode chamber, the cell with commercial N_2_ gas and 5% O_2_ show a measured open circuit voltage of 0.113 V and 0.034 V, respectively, corresponding to a moderate P_O2_ of 10^–3^ and 10^–1.3^ Pa. Excitingly, treating catalyst with N_2_ and 5% O_2_ also leads to the obvious desorption of oxygen ions (as molecular oxygen, confirmed by online mass spectroscopy (MS) data shown in Figure S6), which further triggers the reduction of Pd cation. After treatment in N_2_ for example, the XRD peak of (110) plane of 10Ce‐LFP and LFP shifts from 32.23° to 32.2° and from 32.19° to 32.18°, respectively, and the cell volume increases from 242.93 Å^3^ to 243.19 Å^3^ according to the Rietveld refinement results in Table S1, indicating the expansion of lattice volume due to loss of oxygen ions.^[^ ^25^ ^]^ The formation of surface oxygen vacancies was also directly evidenced by O1s XPS data in which the concentration of surface adsorbed oxygen species increases from 32% to 37% (Figure S7). Figure 1D shows an aberration‐corrected transmission electron microscopy (AC‐TEM) image with the corresponding fast Fourier transformed (FFT) pattern of the 10Ce‐LFP‐N_2_ catalyst, where lattice distances of 0.278 and 0.385 nm are observed, consistent with those of (121) and (101) planes of orthorhombic perovskite. Meanwhile, the bright spots of Pd clusters (yellow circles) with a diameter <2 nm are also found imbedded in the perovskite matrix. Figure 1E further shows the HAADF‐STEM and EDX mapping images of the 10Ce‐LFP‐N_2_. Differently, only homogeneous distribution of the constituent elements of La, Ce, Fe, and O is displayed with the slight segregation of Pd cluster (<2 nm) within the resolution limitation of the instrument. These microscopy images collectively affirm the unique role of N_2_ treatment to selectively trigger the formation of small Pd nanocluster only.

### 2.2 Electronic configuration of reactive Pd species

**FIGURE 2:**
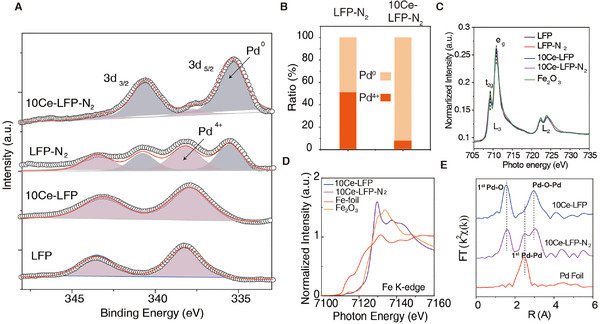
The electronic configuration of various catalysts. (A) Pd 3d XPS spectra of various catalysts. (B) The ratio of Pd species in LFP‐N_2_ and 10Ce‐LFP‐N_2_ catalysts. (C,D) The Fe L‐edge and K‐edge XANES spectra of various catalysts. (E) R‐spaced FT‐EXAFS of LFP‐N_2_ and 10Ce‐LFP‐N_2_ catalysts with the reference of Pd foil

The chemical state of Pd species after N_2_ treatment was then comprehensively analyzed by X‐ray photoelectron spectroscopy (XPS). The untreated LFP and 10Ce‐LFP catalysts possess one single Pd 3d_3/2_ binding energy peak at ∼338 eV, corresponding to the Pd^4+^ species in PdO_6_ octahedra in perovskite (Figure 2A). While treated with N_2_, the 10Ce‐LFP catalyst obtain a major proportion of Pd^0^ species (over 90%), suggesting the nearly complete transformation of cation to metal. This valence change is very similar to the counterpart treated by the H_2_ atmosphere (Figure S8). The as‐formed metallic Pd species deposited on the surface of catalyst was also confirmed by CO FT‐IR spectra in which the bridge absorption of CO on metallic Pd at 1930 cm^–1^ was clearly observed on the 10Ce‐LFP‐N_2_ catalyst (Figures S9 and S10). After fitting the XPS spectra of La3d, Ce3d, and Pd 3d, the Pd/La+Ce ratios of 10Ce‐LFP before and after N_2_ treatment, it can be observed the ratio increased from 3.7% to as high as 8.5% (Table S2), indicates the enhancement in the surface Pd content after N_2_ treatment. Meanwhile, the XPS data further discloses that only 50% of Pd^4+^ was reduced to Pd^0^ on the LFP catalyst (Figure 2B), indicating that the existence of Ce facilitates the reduction of Pd species. The data from XPS is consistent with the H_2_─O_2_ titration experiment (Figure S11) that a larger quantity of metallic Pd emerged on the Ce‐doped catalysts (Figure S11). Exact valence state of Fe ion was checked by soft x‐ray XAS at the Fe‐L_2,3_ edge^[^ ^26^ ^]^ and hard XAS at the Fe‐K edge.^[^ ^27^ ^]^ Excitingly, the Fe valence and coordination status were maintained well (overlapped curve of Fe L‐edge and K‐edge XAS spectra) contaminant with the reduction of Pd (Figure 2C,D), suggesting that the exsolution occurring in N_2_ has high selectivity toward Pd cation only. To verify the coordination information of the Pd cluster on 10Ce‐LFP‐N_2_, the Fourier transform extended X‐ray absorption fine structure spectra (FT‐EXAFS, r space) was carried out, and the profiles are shown in Figure 2E. The existence of Pd‐O coordination (1.6 Å) in the 10Ce‐LFP sample (blue line) can be clearly observed with reference to Pd foil (red line). The Pd‐Pd (2.45 Å) coordination representing the nature of metallic Pd bulk can be detected on the Pd foil curve.^[^ ^28^ ^]^ Interestingly, we found an obvious Pd‐Pd (2.48 Å) coordination in the 10Ce‐LFP‐N_2_ sample, validating the existence of metallic Pd NPs after exsolution. Also, a lower Pd‐O FT intensity can be found in the 10Ce‐LFP‐N_2_. This result authorizes that the N_2_ treated catalyst has a lower coordination number due to the formation of oxygen vacancies.

### 2.3 Catalytic activity measurement

**FIGURE 3:**
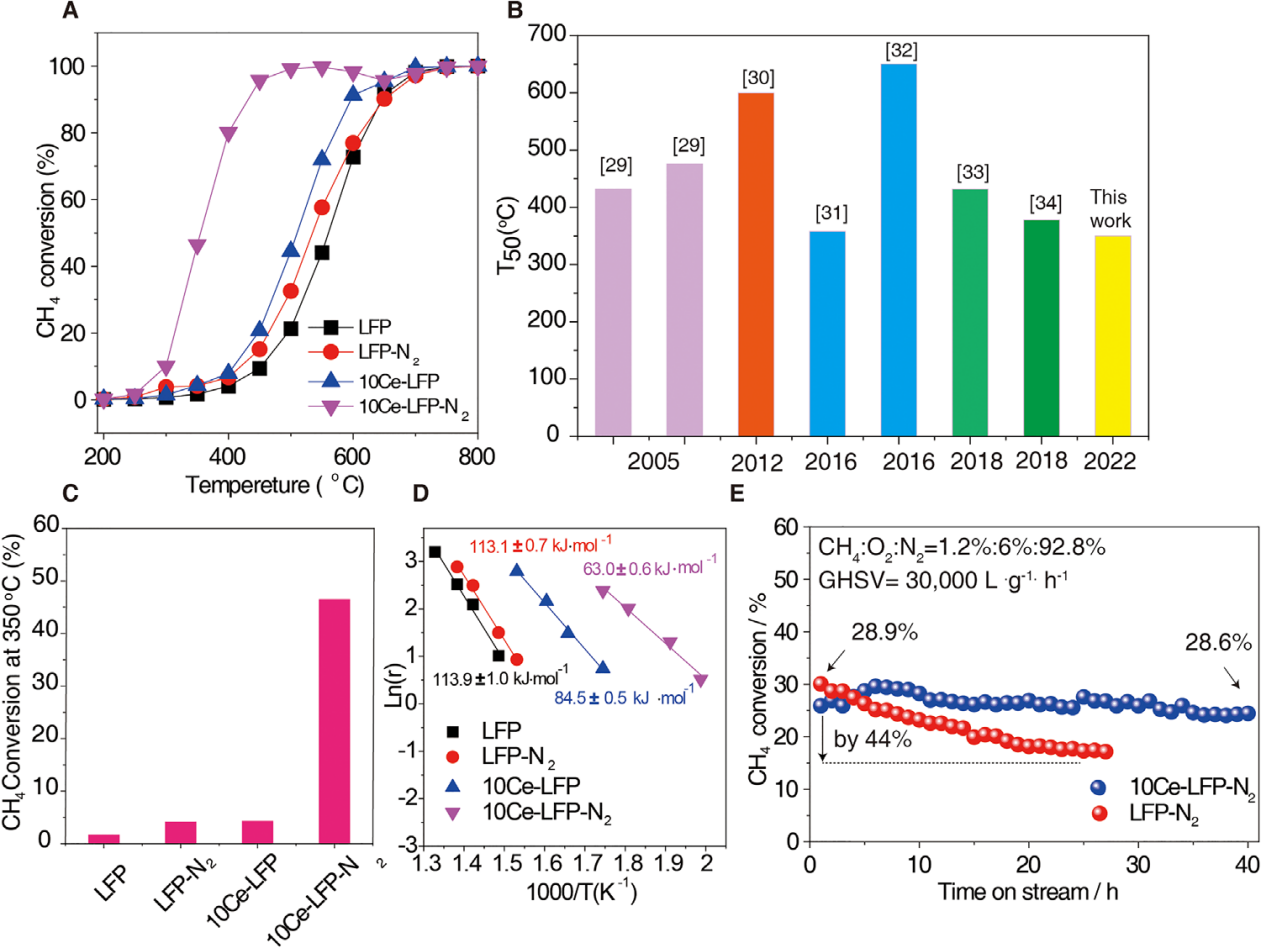
The catalytic activity measurement toward lean methane combustion. (A) Light‐off curves of methane conversion of various catalysts. (B) The comparison of T_50_ of representative oxide catalyst systems.^[^ ^29^, ^30^, ^31^, ^32^, ^33^, ^34^ ^]^ (C) The CH_4_ conversion of different catalysts at 350°C. (D) The calculated activation energy. (E) The stability test of 10Ce‐LFP‐N_2_ and LFP‐N_2_ catalysts at 350°C, *m*_10Ce‐LCF‐N2_ = 100 mg, *m*_LFP‐N2_ = 300 mg

The catalytic activity of CH_4_ combustion curves over various catalysts depending on temperature is shown in Figure 3A. The T_50_ (temperature corresponding to 50% methane conversion) is an important index to compare the performance of catalysts in methane combustion. The parallel comparison of catalysts with different Ce concentrations before and after N_2_ treatment made a quick screen to optimize Ce doping level. The Ce concentration vs. catalytic activity plot demonstrates a volcano‐shaped relationship and the LFP, LFP‐N_2_, 1Ce‐LFP‐N_2_, 5Ce‐LFP‐N_2,_ 10Ce‐LFP‐N_2,_ 20Ce‐LFP‐N_2_ and 30Ce‐LFP‐N_2_ catalyst presents a T_50_ at 560, 534, 485, 401, 350, 381 and 386°C (Figures S12 and S13), suggestive of the outstanding catalytic activity of catalysts incorporated with 10 mol% Ce. Meanwhile, the catalytic activity and P_O2_ also follow a volcano‐like relationship with the one treated with N_2_ demonstrating the best activity (Figure S14). Specifically, the additive of Ce significantly promoted the methane combustion activity since the LFP‐N_2_ catalyst only showed a T_50_ of 534°C. The controlled trial further proves that the motif of Ce in catalyst also plays a determining role in catalytic performance, and the catalyst with impregnated Ce species (10Ce/LFP‐N_2_) has a high T_50_ of 530°C (Figure S15). The activity of 10Ce‐LFP‐N_2_ is comparable to those of highly efficient Pd catalysts and better than other representative perovskite‐based catalysts reported in the past decade (Figure 3B). The Brunner−Emmet−Teller (BET) measurement (Figure S16) reveals that all as‐prepared catalysts obtain similar pore structure and BET surface area, which minimizes the potential influence of physical properties on the evaluation of the intrinsic methane combustion activity of reactive sites.

The CH_4_ conversions at 350°C of Ce (un)doped LFP are shown in the Figure 3C. N_2_ treated 10Ce‐LFP reveals the optimal conversion of CH_4_ (nearly up to 45%). The kinetic investigations were conducted to explore the fundamental difference of intrinsic catalytic activity over these catalysts. Figure 3D shows the temperature‐dependent Arrhenius plots of the CH_4_ conversion rate (r). The determination coefficient (R^2^) for these Arrhenius plots is at least 0.99 to assure the reliability of the data. The 10Ce‐LFP‐N_2_ discloses an apparent activation energy of 63.0  ± 0.6 kJ mol^–1^ which is much lower than other samples. In general, the lower apparent activation energy for catalysts is more likely to motivate methane molecules to be activated,^[^ ^35^ ^]^ which is agreed with the high catalytic activity for 10Ce‐LFP‐N_2_. Besides, the 10Ce‐LFP‐N_2_ catalyst also exhibits excellent stability for prolonged measurement. After 40 h stability tests were performed at 350°C, the catalyst only shows a slight conversion decline from 28.9% to 28.7% which is much more robust than LFP‐N_2_ (decline by 44%), once again confirming its high activity stability (Figure 3E).

### 2.4 Atomic insight into the role of Ce

**FIGURE 4:**
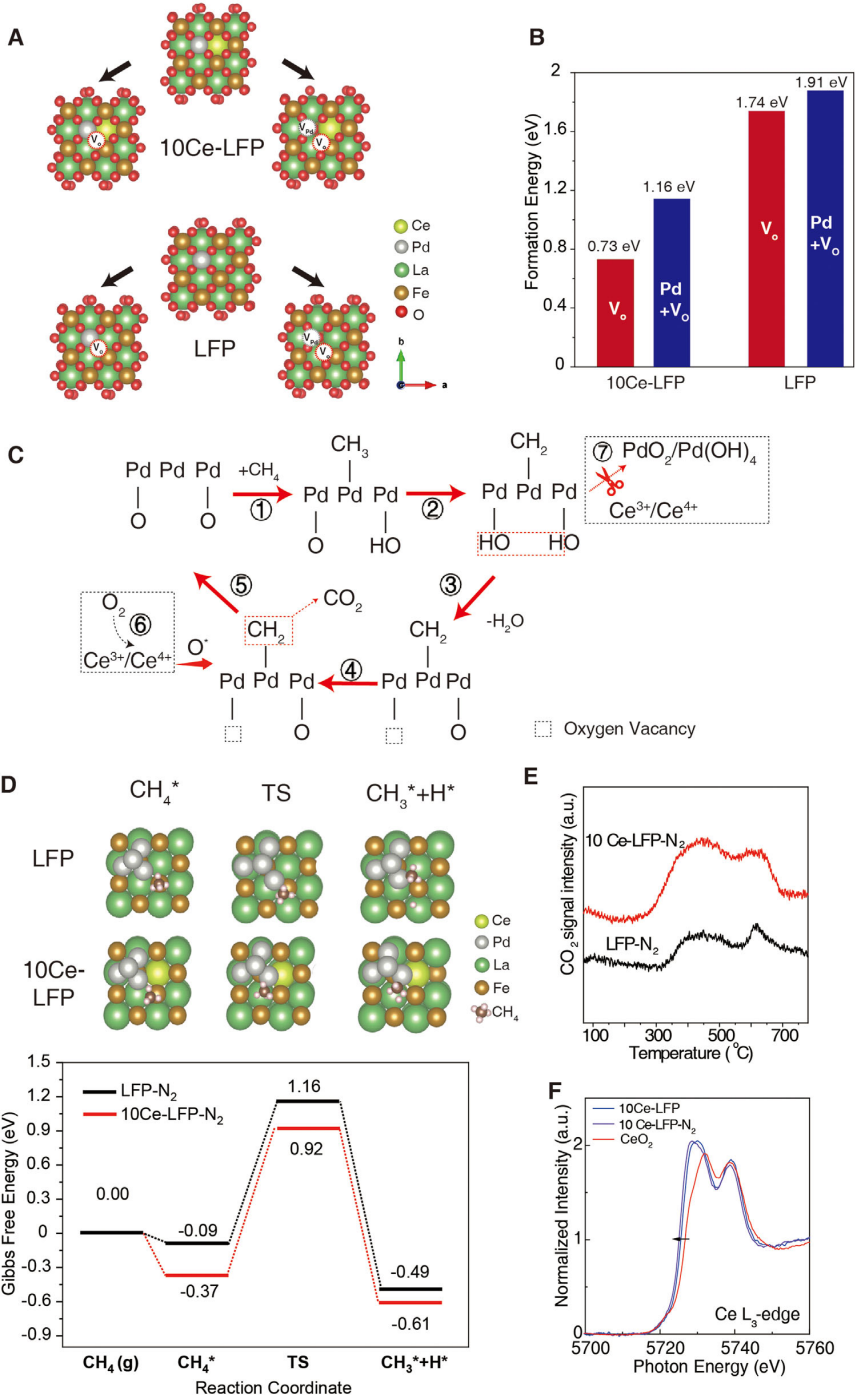
Exsolution and CH_4_ oxidation reaction mechanism. (A) The models used for calculating oxygen vacancies (*V*_o_) formation, Pd, and O co‐segregation energy. (B) The Comparison of the Vo formation energy and co‐segregation energy. (C) The proposed CH_4_ oxidation reaction mechanism. (D) Top view of structures for adsorbed CH_4_ (CH_4_^*^), TS, and co‐adsorbed CH_3_ and H (CH_3_* + H^*^) on PdO(101) and Calculated free energy diagrams for breaking the first C─H bond in CH_4_ on (010) surface. The Gibbs free energy values were calculated using a temperature of 350°C. (E) CO_2_ TPD profile. (F) Ce‐L_3_ edge XANES of various catalysts

The experimental results shown above have unambiguously illustrated the unique role of Ce additive in A‐site with multi‐dimension manipulation effects. To get atomic insights into how Ce affects the construction of the catalytic platform, the DFT simulation was carried out to simulate the exsolution process of Pd. As discussed above, the exsolution process typically includes two major stages, that is, (1) the formation of oxygen vacancies, (2) diffusion/segregation of metallic Pd. Kwon et al. studied the co‐segregation energy of a series of transition metal‐doped layered perovskite, including the formation energy of metal and oxygen vacancies,^[^ ^36^ ^]^ and pointed out that the creation of oxygen vacancies is a prerequisite step for exsolution. Accordingly, the reactivity of oxygen ions adjacent to PdO_6_ was preliminarily investigated. 10Ce‐LFP obtains a closer O 2p vs fermi level distance of −2.7 eV than that of LFP (−2.8 eV) (Figure S17), and the formation free energy (*ΔG*) of a single oxygen vacancy from PdO_6_ on 10Ce‐LFP is 0.73 eV which is evidently smaller than that on LFP crystal (1.74 eV) (shown in Figure 4A,B). This result implies the higher mobility of surface oxygen ions after the incorporation of Ce, which is consistent with XPS results (Figure S7). The co‐exsolution (Pd+O) energy of 10Ce‐LFP (1.16 eV) is also much lower than the counterpart without Ce (1.91 eV) (Figure 4B), and the Pd formation energy from perovskite bulk is also thermodynamically favorable (Figure S18), indicative of the benefited overall exsolution process after Ce doping.

Besides the facilitation of ultra‐fine Pd cluster, the Ce tightly couples with Pd species, collectively participating in the methane conversion. It is universally accepted that the combustion reaction of CH_4_ catalyzed by Pd catalysts often follows the classical Mars‐van Krevelen (MvK) mechanism^[^ ^37^, ^38^ ^]^ in which the lattice oxygen plays a key role in the activation of CH_4_. The pulse experiment of CH_4_ confirms the significant conversion of CH_4_ pulse by lattice oxygen which can be refilled by consecutive O_2_ pulse (Figure S19). Furthermore, it is commonly acknowledged that the transformation between PdO and Pd generates the metastable intermediates in the redox cycle.^[^ ^5^, ^39^ ^]^ Typically, CH_4_ is preferentially adsorbed on the Pd/Pd–O interface to yield a –CH_3_ radical and a –OH (CH_4_ + Pd/Pd–O → Pd–CH_3_/Pd–OH, step ① in Figure 4C),^[^ ^15^, ^40^ ^]^ and another H atom from the cracking of –CH_3_ on a neighboring Pd–O is sequentially incorporated to form an adjacent Pd–OH (step ② in Figure 4C). Then the two absorbed –OH are combined to yield one H_2_O and one Pd site (2Pd–OH →H_2_O + Pd–O + Pd) (step ③ in Figure 4C).^[^ ^41^ ^]^ Also, the hydrocarbon radicals react with the O ions in PdO to form CO_2_ (step ④ in Figure 4C). Simultaneously, PdO that loses few O atoms is subsequently re‐oxidized by oxidative cocatalysts or molecular oxygen to achieve a close reaction loop of PdO→Pd→PdO(step ⑤ in Figure 4C).^[^ ^42^ ^]^ The in situ formed oxygen vacancies on the cocatalyst will be refilled by molecular oxygen in reaction gas (step ⑥ in Figure 4C).^[^ ^43^ ^]^ Based on this mechanism, two key steps will determine the reaction rate, that is, fast CH_4_ activation to form hydrocarbon radicals and effective oxygen exchange to regenerate PdO.

As for the former, the reaction rate of CH_4_ activation is correlated to the d‐orbital filling of Pd species that affects the dissociation of CH_4_ to CH_3_* radical and the further generation of Pd‐OH. Therefore, the adsorption of the CH_4_ state on the mode surface, the transition states (TSs), and the final dissociation states of 10Ce‐LCP and LCP catalysts were computed, respectively, and the corresponding Gibbs free energy diagrams were calculated, as shown in Figure 4D. The DFT calculations were performed on the surfaces containing Pd/Perovskite (010) facet (The mode is shown in Figure S20a). The optimal CH_4_ absorption site is Pd atom bonding to the perovskite surfaces. With the assumed temperature at 350°C, the Gibbs free energy of adsorbed methane on the surface with/without Ce is −0.09 and −0.37 eV, respectively (The potential energy profiles are also shown in Figure S20b). These values indicate that CH_4_ binds more strongly to the surface containing Ce in the lattice. The TS Gibbs free energy (energy difference between C─H cleavage barrier and desorption barrier of CH_4_) is 1.16 eV and 0.92 eV for 10Ce‐LFP‐N_2_ and LFP‐N_2_, respectively. Thereby, the computational results evidence that the additive of Ce could benefit the C–H activation on the metal/perovskite interface.

On the other hand, the total reaction rate was also affected by the migration/exchange rate of oxygen to maintain the stoichiometry of PdO to offset its continuous decomposition. Previous studies have provided detailed insight into the regeneration process of PdO, and found that O exchange of the active PdO species can be suppressed by adsorbed –OH species via the generation of Pd(OH)_4_ and/or PdO_2_, thus deactivating the catalyst.^[^ ^39^, ^44^ ^]^ Fortunately, it is found that the increased basicity of catalyst not only can inhibit the accumulation of –OH on the active sites,^[^ ^45^, ^46^ ^]^ but also can facilitate the formation of PdO. Usually, the CeO_2_ can be an ideal source offering basicity for diverse metal/oxide catalyst systems.^[^ ^47^, ^48^, ^49^ ^]^ In our study, the presence of the Ce element among the perovskite frame also endows the catalyst with extra basic sites without the formation of isolated CeO_2_ crystal, which was proved by CO_2_‐TPD (Figure 4E). The peak at around 400°C can be attributed to weak physically adsorbed CO_2_, and the peak at around 600°C is associated with CO_2_ adsorption on strong basic sites.^[^ ^50^ ^]^ Clearly, the intensities of both peaks are much stronger on 10Ce‐LFP samples, confirming the supply of sufficient base sites from Ce to inhibit the accumulation of surface –OH (step ⑦ in Figure 4C).^[^ ^51^ ^]^ Meanwhile, as illustrated by Ce L_3_‐edge XANES (Figure 4F), the average oxidative state of Ce in 10Ce‐LFP‐N_2_ is significantly lower than that of CeO_2_ by shifting the absorption edge to the lower binding energy region by around 1.5 eV, and also slightly lower than that of 10Ce‐LFP. The XPS result also discloses that the quantity of Ce^3+^/Ce^4+^ pairs was boosted by ∼81.3% (from 20.2% to 36.3%) after N_2_ treatment (Figure S21), which is expected to chemically absorb and react with more gaseous O_2_ and promote the oxygen exchange during the reaction (step ④ in Figure 4C).^[^ ^42^ ^]^ Finally, the XPS measurement was employed again to study the real oxidative state of Pd species after a prolonged test. As shown in Figures S22 and S23, the catalyst modified with Ce exhibits strong prohibition against the deep oxidation and obtains a combination of highly active Pd species of Pd^2+^ (existence of imbedded PdO cluster shown in Figure S23) and Pd^0^, corresponding to its better performance. By contrast, the LFP without Ce shows the large proportion of deeply oxidized Pd species of Pd^4+^, leading to poor activity.

## 3 CONCLUSION

In summary, we have reported that the facile amelioration (using N_2_) of traditional in situ exsolution strategy could successfully guide the fabrication of highly performed catalyst containing emergent Pd cluster anchored into perovskite parent. The as‐prepared catalyst delivers a T_50_ as low as 350°C toward trace methane oxidation and a stable conversion for over 30 h. Further, we have reported experimentally and theoretically that the atomically dispersed Ce in perovskite lattice exhibits exceptional multi‐dimension tunability on both exsolution and reaction. Briefly, the existence of Ce in perovskite matrix thermodynamically and kinetically benefits the emergence of metallic Pd phase, which also lowered the energy barrier of the C─H bond cleavage. More importantly, by serving as the oxygen reservoir involved into MvK pathway, Ce can prohibit the deep oxidation of Pd and help maintain a suitable ratio of Pd^2+^/Pd^0^ as a reactive couple. We believe the findings in this work provide a valuable example to design high‐performance combustion catalysts.

## 4 EXPERIMENTAL SECTION

### 4.1 Catalyst preparation

The La_1−_ *_x_* Ce*_x_* Fe_0.97_Pd_0.03_O_3_ perovskite was fabricated by a sol–gel method. Stoichiometric metal nitrate precursors of La(NO_3_)_3_·6H_2_O (Macklin, >99%), Ce(NO_3_)_3_·6H_2_O (Energy Chemical, 99%), Fe(NO_3_)_3_·9H_2_O (Energy Chemical, 99%) and (NH_4_)_4_Pd(NO_3_)_6_ (Energy Chemical, 99%) with the molar ratio of (100 − *x*):*x*:97:3 were completely dissolved into certain amount of deionized water to prepare an aqueous metal salt solution of A (0.05 mol · L^–1^). After that, the citric acid was added and completely dissolved into A to make solution of B (the total metal ions/citric acid (molar ratio) is kept at 1:2). The B solution was stirred at 80°C until the honey‐comb like gel was formed. After that, the gel was kept in an oven overnight at 100°C. The obtained material was finely grounded and calcined at 800°C in a furnace for 4 h with a rate of 5°C · min^–1^ to form crystallinity. After natural cooling, the as‐obtained brown powder was denoted as *x*Ce‐LFP (*x* = 0, 1, 5, 10, 20, 30, responding to the percentage of Ce element in A‐Site).

Upon in situ exsolution procedure, the as‐prepared catalysts were annealed in different atmospheres at elevating temperature and the typical process was shown as follows: a certain mass of catalyst was placed in a crucible and heated to 200°C with a ramping rate of 5°C · min^–1^ from room temperature in the atmosphere of 5%H_2_/N_2_, pure N_2_, 5%O_2_/N_2_ and air (the flow rate is 50 ml · min^–1^), respectively. After the pre‐treatment of 30 min, the catalyst was annealed to 850°C at a heating rate of 3°C min^–1^. Then natural cooling to room temperature, the as‐gained samples were denoted as *x* (Ce)‐LFP‐H_2_, *x* (Ce)‐LFP‐N_2_, *x* (Ce)‐LFP‐O_2_, and *x* (Ce)‐LFP‐Air, respectively.

### 4.2 Material characterizations

Powder XRD patterns of all catalysts were performed on a Panalytical X'pert PRO diffractometer (Philips, Netherlands) with the Cu Kα radiation (*λ* = 0.15406 nm) at a voltage of 40 kV and a current of 30 mA. The Rietveld refinement analysis was performed by using a EXPGUI GSAS software by the means of ordinary least squares based on the raw XRD data.

H_2_‐temperature‐programmed reduction (H_2_‐TPR) was performed on a homemade fixed‐bed reactor. 100 mg of the catalyst was placed in the quartz tubing reactor plugged with quartz cotton at both ends and H_2_‐TPR experiments were then conducted by heating the sample from 30 to 850°C (rate of 5°C · min^–1^) in a flowing gas of 5% H_2_/Ar (20 ml min^–1^). The H_2_ uptake was measured and quantified using a thermal conductivity detector (TCD).

The measurement of oxygen partial pressure (P_O2_) was obtained on a homemade solid oxide concentration cell conducted by the CS2350H (Corrtest, China) electrochemical workstation. The assembling procedure of the cell can be found as follows: The electrolyte of 8 mol% yttrium‐doped zirconia (YSZ, 300 μm in thickness and 2.5 inches in diameter, fuelcellmaterials.com) supported single solid oxide cell with configuration of (Pt impregnated porous YSZ)/YSZ/(Pt impregnated porous YSZ) were constructed for monitoring the P_O2_. The screen‐printing technique was used to fabricate electrolyte‐supported cells. The anode and cathode YSZ (Xuzhou Huaqing) ink with an area of 0.5 cm^2^ was screen painted on both sides of YSZ electrolyte and annealed in air at 1100°C for 5 h. The ethyecellulose and terpilenol in electrode ink were burnt to form porous structure. The 1 wt% Pt (chloroplatinic acid, Macklin) was infiltrated into porous YSZ matrix and annealed at 600°C for 4 h. Current collector for cell test is acted by a high temperature silver paste, which was screen‐printed on electrode surface. Finally, the ceramic‐based sealant (Cerama‐bond 552, Aremco) was used to seal the cells on an alumina tube. The detail of the measurement procedure is shown in Figure S4.

Temperature‐programmed desorption experiments in N_2_, 5%O_2_/N_2_, or air atmospheres were conducted on the catalyst with a dosage of 200 mg to detect the oxygen loss during exsolution. Specifically, for the measurement in N_2_, the reactor was connected to an Omnistar^TM^ online mass spectrometer (MS, Pfeiffer vacuum, USA) instrument. And the sample was heated from 30 to 800°C with a ramping rate of 5°C · min^–1^ in flowing N_2_ (20 ml · min^–1^), and kept at 800°C for another extra hour. The O_2_ (*m*/*z* = 32) signal was monitored online. While in 5%O_2_/N_2_ and air atmospheres, the measurement was carried out from 30 to 950°C with the ramping rate of 5°C · min^–1^, and the GC equipped with TCD (carrier gas of 5%O_2_/N_2_ and air, respectively) was applied to detect the variation of oxygen content.

The XPS characterization was performed on the ESCALAB™ XI^+^ instrument (ThermoFisher, USA) with Al Kα irradiation as the excitation source at 5.0 × 10^–9^ mbar. All binding energy values were standardized according to C 1s signal at 284.8 eV. The deconvolution of XPS data was performed using Peak41 software.

X‐ray absorption near‐edge structure (XANES) characterization for O K edge and Fe L edge were collected at the Beamlines MCD‐A and MCD‐B (Soochow Beamline for Energy Materials) at National Synchrotron Radiation Laboratory (NSRL, China).

To study the morphology of the catalysts, the transmission electron microscope (Tecnai G2 F20 (TEM; FEI, U.S.) and JEM‐ARM200F operating at 200 kV) equipped with energy dispersive X‐ray (EDX) detector were utilized to collect the HAADF‐STEM images and EDX mapping. For preparation of TEM samples, e A small amount of the sample was taken to ethanol solution (99.9%, Sinopharm chemical) and sonicated for 30 min. The carbon‐coated copper mesh (400 mesh, Electron Microscopy China) was dipped into dispersed slurry for three times, and dried in an oven for 2 h.

A Nicolet iS50 (ThermoFisher, USA) spectrometer equipped with a HVC‐MRA‐5 reaction chamber (Harrick) and MCT detector was employed to collect the Diffuse reflectance infrared Fourier transform (DRIFT) spectroscopy. The samples were dried at 105°C for an hour in N_2_ (50 ml · min^–1^) before the CO adsorption and then naturally cooled down. The IR signal was collected after 5%CO/N_2_ (20 ml · min^–1^) was introduced to the reaction chamber based on the background spectrum taken in N_2_ atmosphere. After being kept for 30 min, the CO flow was replaced by N_2_ flow and desorption was followed over 20 min.

The N_2_ isothermal adsorption and desorption were performed at −196°C using an automated Micromeritics ASAP2020 Plus instrument (Norcross, USA).

CO_2_‐temperature programmed desorption (CO_2_‐TPD) was performed on a Hiden Analytical QIC‐20 instrument equipped with a mass spectrometer. Before the TPD measurement, 0.1 g samples were placed in the center of the middle of the quartz tube, and then injected He (30 ml min^–1^) to purge the sample at 300°C for 1 h with a 5°C · min^–1^ heating rate and then cooled to 30°C. Then the 10% CO_2_/He mixture gas (50 ml min^–1^) was introduced to act as the adsorbate at 30°C. After adsorbing for 0.5 h, the He (30 ml min^−1^) was injected to flush the physical adsorption of CO_2_ or CO_2_ in the gas phase. Meanwhile, the MS was launched to detect the signal of CO_2_ until the baseline was stable. Finally, temperature‐programmed desorption was performed from 30 to 800°C at 10°C min^–1^.

H_2_‐O_2_ titration experiment was performed following the procedure: 100 mg sample was placed in a U‐shaped quartz tube and swept with N_2_ at the flow rate of 50 ml min^–1^. The temperature was raised to 300°C with a constant rate for 30 min, and kept for 1 h to clean the surface. And then, the gas was switched to 5%H_2_/N_2_ (50 ml min^–1^) for 1 h to completely reduce the reducible cations to metal. According to H_2_‐TPR result, the Fe cation is stable at this low temperature. After that, the N_2_ was purged for another 1 h and the sample was cooled down to 100°C. And then, the O_2_ was introduced for 1 h at a flow rate of 30 ml min^–1^. Finally, the sample was purged with N_2_ for 1 h. After this multi‐step pretreatment, the 1 ml quantitative loop fulfilled with H_2_ in the system was pulsed into the U‐shape tubing to perform hydrogen titration of the oxygen atoms adsorbed chemically. Each hydrogen outflow signal was recorded by the mass spectrum (Hiden Analytical QIC‐20 instrument equipped with a mass spectrometer).

### 4.3 Catalytic activity testing

The methane conversion performances (light‐off test) of all catalysts were measured on a fixed‐bed flow reactor with an inside diameter of 5 mm. A K‐type thermocouple was placed in the middle region of the reactor. 100 mg of catalysts were placed at the center of the tube, which were plugged with quartz cotton at both ends. The quartz tube was placed in a tube furnace. The products were analyzed by a gas chromatograph (GC, Ruimin 2060 GC) equipped with a hydrogen flame ionization detector (FID). All samples were pretreated in an atmosphere of 5% O_2_/N_2_ at 200°C for 2 h before measurement. After that, pure N_2_ was introduced to sweep the sample for 0.5 h. And then the mixed reaction gas (1.2 vol% and CH_4_, 6 vol% O_2_ balanced N_2_) was introduced with the total flow rate of 50 ml/min (gas hourly space velocity (GHSV) of 30,000 ml∙g_cat_^–1^∙h^–1^). The GC data were collected every other 50°C from 200 to 850°C. Before collecting the data at any specific temperature, the catalyst was stabilized for at least 15 min.

The conversion of CH_4_ was calculated according to the following equation (Equation (2)):

(2) $$CH_{4}\;\mathrm{conversion}\left(\%\right)=\left(CH_{4,\mathrm{in}}-CH_{4,\mathrm{out}}\right)/CH_{4,\mathrm{in}}\times 100$$

The reaction kinetic was studied with CH_4_ conversion lower than 10%, to ensure a kinetically controlled regime and eliminate the thermal and diffusion effects. The reaction rates (r) were calculated by the following equation (Equation (3)):

(3) $${r_{\mathrm{CH}}}_{4}=({C_{\mathrm{CH}}}_{4}\times {X_{\mathrm{CH}}}_{4}\times v\times M_{\mathrm{Pd}})/V_{m}\times m_{\mathrm{cat}}\times w_{\mathrm{Pd}}$$

where *m*_cat_ is the mass of catalyst used for measurement, *w*_Pd_ is the Pd loading amount calculated by initial feeding content, *C*_CH4_ is the volume ratio of CH_4_ in the feed gas, *v* represents flow rate of mixture gas, *V*_m_ is the molar volume of gas, *X*_CH4_ is the conversion of CH_4_, *M*_Pd_ stands for the atomic weight of Pd (106.4 g·mol^–1^)

### 4.4 Computational calculations

The DFT calculations were performed using a plane‐wave basis set in the Vienna Ab‐initio Simulation Packages (VASP 5.4.1).^[^ ^52^ ^]^ The Perdew–Burke–Ernzerhoff (GGA‐PBE) functional^[^ ^53^, ^54^ ^]^ with the dispersion correction (Grimme's DFT‐D3)^[^ ^55^ ^]^ was utilized to calculate the exchange and correlation energies. The core electrons were described by using a projected‐augmented wave (PAW)^[^ ^56^ ^]^ pseudopotentials. A cutoff energy of 400 eV for the plane‐wave basis sets was set for all the calculations. For bulk LaFeO_3_, supercells, and surfaces, a Monkhorst‐Pack^[^ ^57^ ^]^ *k*‐point grid of 4 × 4 × 4, 2 × 2 × 1, and 2 × 2 × 1 were adopted, respectively. An on‐site Hubbard term *U*‐*J* was added to address the open‐shell f‐electrons (6.0 eV for La, 4.5 eV for Ce) and d‐electrons (4.0 eV for Fe, 4.0 eV for Pd).^[^ ^58^, ^59^ ^]^ The residual forces and system energies were converged to 0.05 eV Å^−1^ and 10^−4^ eV, respectively.

Based on our experiment data, bulk LaFeO_3_ (*Pnma*, No. 62) was chosen and a 2´2´1 LaFeO_3_ supercell (La_16_Fe_16_O_48_) was built. The optimal lattice parameters with *a* = 5.43 Å, *b* = 5.47 Å, *c* = 7.70 Å were used for LaFeO_3_ cell. The LaFeO_3_ (010) surface was modeled with three Fe‐O and two La‐O layers in a *p*(2 × 2) surface unit cell. In order to avoid interactions between slabs, a vacuum space of 30 Å was added on the surfaces. The slab was built with the two bottommost layers well fixed and top three layers fully relaxed for participation in reaction. Corresponding Fe/La atoms were substituted by Pd/Ce atoms to model the doped catalysts. The Pd_4_ clusters were loaded on the catalyst surface via bonding with lattice oxygen (Pd─O) to simulate in situ exsolution interfacial structures. The elementary reaction barriers and structures of transition states were located using the climbing image nudged elastic band (CI‐NEB) method.^[^ ^60^ ^]^

The oxygen vacancy‐formation energy *E*_vf_(O) was calculated by,

(4) $$E_{\mathrm{vf}}\left(O\right)=\left[E_{\mathrm{vac}}\left(O\right)+\frac{1}{2}E_{O_{2}}\right]-E_{\mathrm{clean}}$$

where *E*_vac_(O) is the system energy with an oxygen vacancy, *E*_O2_ is the total energy of an isolated oxygen molecule in the gas phase, and *E*_clean_ is the total energy of perovskite slab structure.

The Pd‐O vacancy‐formation energy *E*_vf_(Pd‐O) was calculated by,

(5) $$E_{\mathrm{vf}}\left(\mathrm{Pd}-O\right)=\left[E_{\mathrm{vac}}\left(\mathrm{Pd}-O\right)+\frac{1}{4}E_{pd_{4}}+\frac{1}{2}E_{O_{2}}\right]-E_{\mathrm{clean}}$$

where *E*_vac_(Pd‐O) is the system energy with an Pd‐O atoms vacancy, $E_{Pd_{4}}$ is the total energy of an isolated Pd_4_ cluster.

The Gibbs free energy (*T* = 623.15 K) was calculated by

(6) $$G\;=E_{DFT}\;+E_{\mathrm{ZPE}}-TS$$

where *E*_DFT_ is the total energy calculated by DFT at 0 K, *E*_ZPE_ is the zero‐point energy, and *S* is the energy of entropy. The vibrational frequency calculations were employed to gain the values of *E*_ZPE_ and *S*.^[^ ^61^ ^]^ During calculations, real frequencies below 50 cm^−1^ are raised to 50 cm^–1^ for simulating low‐frequency anharmonic effects.^[^ ^62^ ^]^ For CH_4_, the treatment is the same except *S* was taken from the NIST database.

The Gibbs free energy change ($\Delta G^{‡}$) of a reaction on the surface can be obtained by:

(7) $$\Delta G^{‡}=G_{transition}{\;}_{State}-G_{\mathrm{reactant}}$$

where *G*_transition state_ is the Gibbs free energy of transition state, *G*_reactant_ is the Gibbs free energy of reactant.

The adsorption free energy (Δ*G*_ads_) of CH_4_ is calculated by

(8) $$\Delta G_{ads}=G_{C{H_{4}}^{*}}-G_{*}-G_{CH_{4}}$$

where *G*_CH4*_ is the Gibbs free energy of a CH_4_ adsorbed on the catalyst, G_*_ is the Gibbs free energy of a clean catalyst, and *G*_CH4_ is the Gibbs free energy of a gaseous CH_4_.

## CONFLICT OF INTEREST

The authors declare no conflict of interest.

## Supporting information



Supplementary Material

## Data Availability

All data associated with this study are present in the paper or in the Supporting Information.
