# Supplementary material for: Atomic cerium modulated palladium nanoclusters exsolved ferrite catalysts for lean methane conversion
Source: Exploration (Beijing). 2022 Jul 11;2(6):20220060. doi: 10.1002/EXP.20220060 (PMC10190994; doi:10.1002/EXP.20220060)
Supplement: Supplementary file 1 — Supplementary Material [file EXP2-2-20220060-s001.docx]

Supplementary Material

**Atomic Cerium modulated Palladium nanoclusters exsolved ferrite catalysts for lean methane conversion**

Yanling Yang^a, 1^, Si Wang^c, 1^, Xin Tu^d, *^ Zhiwei Hu^e^, Yinlong Zhu^f,*^, Hongquan Guo^a^, Zhishan Li^a^, Li Zhang^a^, Meilan Peng^a^, Lichao Jia^g^, Meiting Yang^h^, Guangming Yang^h^, Xurong Qiao^i^, Jiahui Sun^i^, Xiaolu Liang^i^, Zhen Zhang^i^, Yanru Zhu^c^, Lei Shi^j^, Chenxing Jiang^a^, Yingru Zhao^a^, Jianhui Li^k^, Zongping Shao^h^, , Xin Zhang^c, *^, and Yifei Sun^a, b, l *^

^a^ College of Energy, Xiamen University, Xiamen 361005, China

^b^ State Key Laboratory of Physical Chemistry of Solid Surface, Xiamen University, Xiamen 361005, China

^c^ Beijing State Key Laboratory of Chemical Resource Engineering, Beijing Advanced Innovation Center for Soft Matter Science and Engineering, Beijing University of Chemical Technology, Beijing 100029, China

^d^ Department of Electrical Engineering and Electronics, University of Liverpool, Liverpool L69 3GJ, UK

^e^ Max Planck Institute for Chemical Physics of Solids, Dresden 01187, Germany

^f^ Institute for Frontier Science, Nanjing University of Aeronautics and Astronautics, Nanjing 210001, China；

^g^ School of Materials Science and Engineering, State Key Lab of Material Processing and Die & Mould Technology, Huazhong University of Science and Technology, Wuhan 430074, China

^h^ State Key Laboratory of Materials-Oriented Chemical Engineering, College of Chemical Engineering, Nanjing Tech University, Nanjing, 211816, China

^i^ State Key Laboratory for Mechanical Behavior of Materials, Xi’an Jiaotong University, Xi’an 710049, China

^j^ School of Chemical Engineering, Dalian University of Technology, Dalian 116024, China

^k^ National Engineering Laboratory for Green Chemical Productions of Alcohols-Ethers-Esters, College of Chemistry and Chemical Engineering, Xiamen University, Xiamen 361005, China

^l^ Shenzhen Research Institute of Xiamen University, Shenzhen, Guangdong, 518057, China

**^*^ Corresponding authors.**

E-mail: zhuyl1989@nuaa.edu.cn(Y. Zhu)；xin.tu@liverpool.ac.uk (X. Tu) ；zhangxin@buct.edu.cn (X. Zhang) ；yfsun@xmu.edu.cn (Y. Sun)

^1^ These authors contributed equally: Yanling Yang, Si Wang


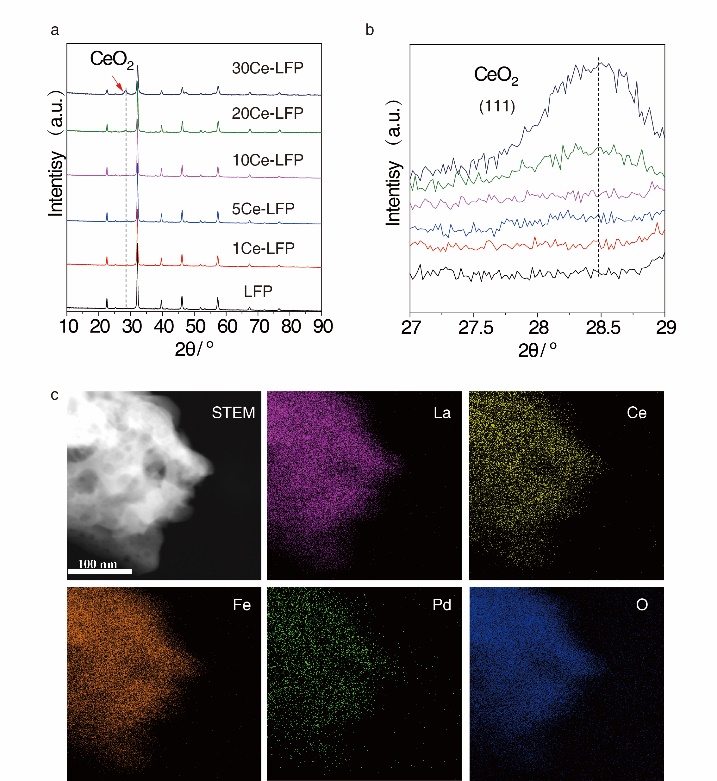


**FIGURE S1** Crystallinity and morphology of Ce doped LFP catalysts. a, The XRD pattern of LFP catalysts with different Ce doping levels. b, The zoom-in XRD pattern ranging from 2θ=27- 29^o^ of various catalysts. As the Ce content raises over 10 mol%, the diffraction peak belonging to (111) plane of CeO_2_ at 28.5 ^o^ can be observed on 20 Ce-LFP and 30 Ce- LFP catalysts, implying that the doping of Ce into A-site of perovskite has a saturation level of 10 mol% c, The HAADF-STEM and EDX mapping images of 10Ce-LFP catalyst. Each element (La, Fe, Ce, Pd and O) is evenly distributed, suggesting the formation of pure phase without secondary phase.


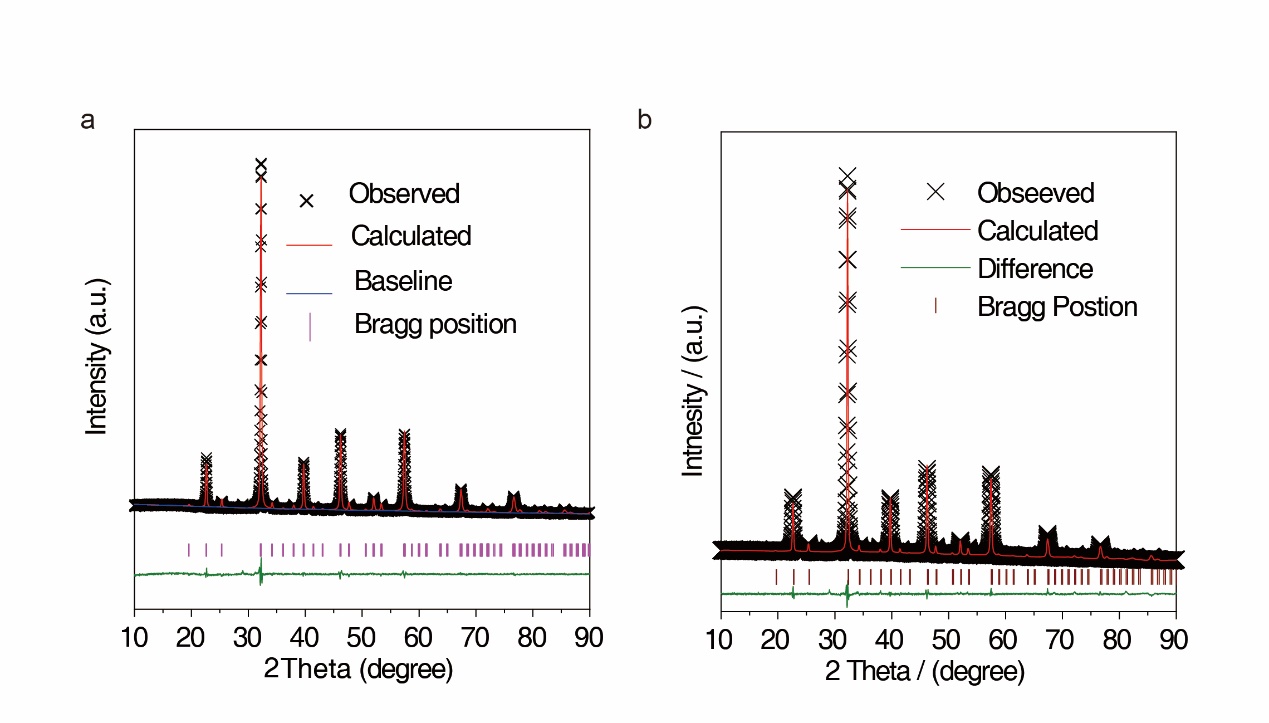


**FIGURE S2** The Rietveld refinement of XRD plot of (a)10Ce-LFP and (b) 10Ce-LFP-N_2_ catalysts. The 10Ce-LFP and 10Ce-LFP-N_2_ catalyst obtain an orthorhombic perovskite structure with the refined lattice parameters shown in Table S1.

**Table S1** The lattice parameters of 10Ce-LFP and 10Ce-LFP-N_2_ catalysts.

| Catalyst | R_wp_(%) | R_p_(%) | χ^2^ | Space group | a(Å) | b(Å) | c(Å) | V(Å^3^) | α(^o^) | β(^o^) | γ(^o^) |
| --- | --- | --- | --- | --- | --- | --- | --- | --- | --- | --- | --- |
| 10Ce-LFP | 6.45 | 5.06 | 4.17 | Pmna | 5.567 | 7.850 | 5.559 | 242.93 | 90 | 90 | 90 |
| 10Ce-LFP-N_2_ | 5.35 | 3.88 | 2.34 | Pmna | 5.561 | 7.864 | 5.561 | 243.19 | 90 | 90 | 90 |


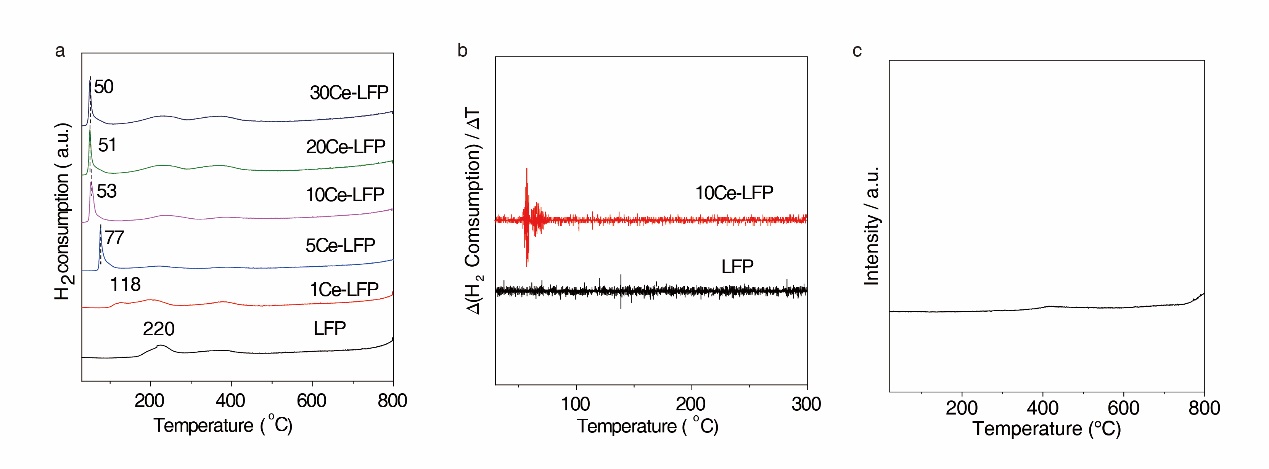


**FIGURE S3** The H_2_-temperature programmed reduction (H_2_-TPR) profile of LFP catalysts with different Ce doping levels. a, TPR profile of various catalysts. The carrier gas is 5 vol% H_2_/N_2_ and the ramping rate is 5 ^o^C min^-1^. Previous work illustrated the reduction peak of LaFeO_3_ has a higher reduction temperature over 300 ^o^C. The pristine LFP catalyst obtains a very broad reduction peak (Pd^4+^ to Pd^2+^ or Pd^0^) starting from 220 ^o^C at which the Fe ion will not be reduced.^[1]^ After incorporating 10 mol% Ce, the reduction peak evolves to a sharp and narrowed one at 53 ^o^C, suggesting that the Ce doping facilitates the reduction of Pd thermodynamically. b, The first derivate plot of TPR profile. The temperature dependent H_2_ consumption rate of as-prepared catalysts is calculated by plotting the Δ(H_2_ consumption)/ Δt vs. Temperature. Obviously the H_2_ consumption rate (intensity of Δ(H_2_ consumption)/ Δt) of 10Ce-LFP is much higher than that of LFP, suggesting that the Ce doping also facilitate the reduction of Pd kinetically. c, TPR profile of LaFeO3 sample with equal vertical scale range.


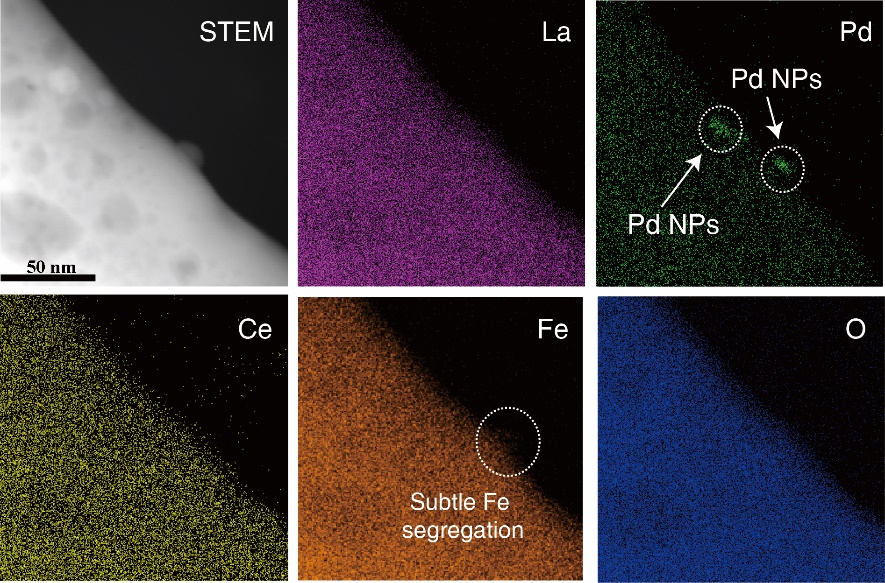


**FIGURE S4** HAADF-STEM and corresponding EDX mapping image of 10Ce-LFP-H_2_ catalyst. The surface exsolution of metallic Pd nanoparticles with the diameter of 15-20 nm can be observed. Besides, subtle Fe segregation was also detected on the surface, suggesting the possible reduction of Fe cations. This result is consistent with the observation shown in previous reference.^[2]^


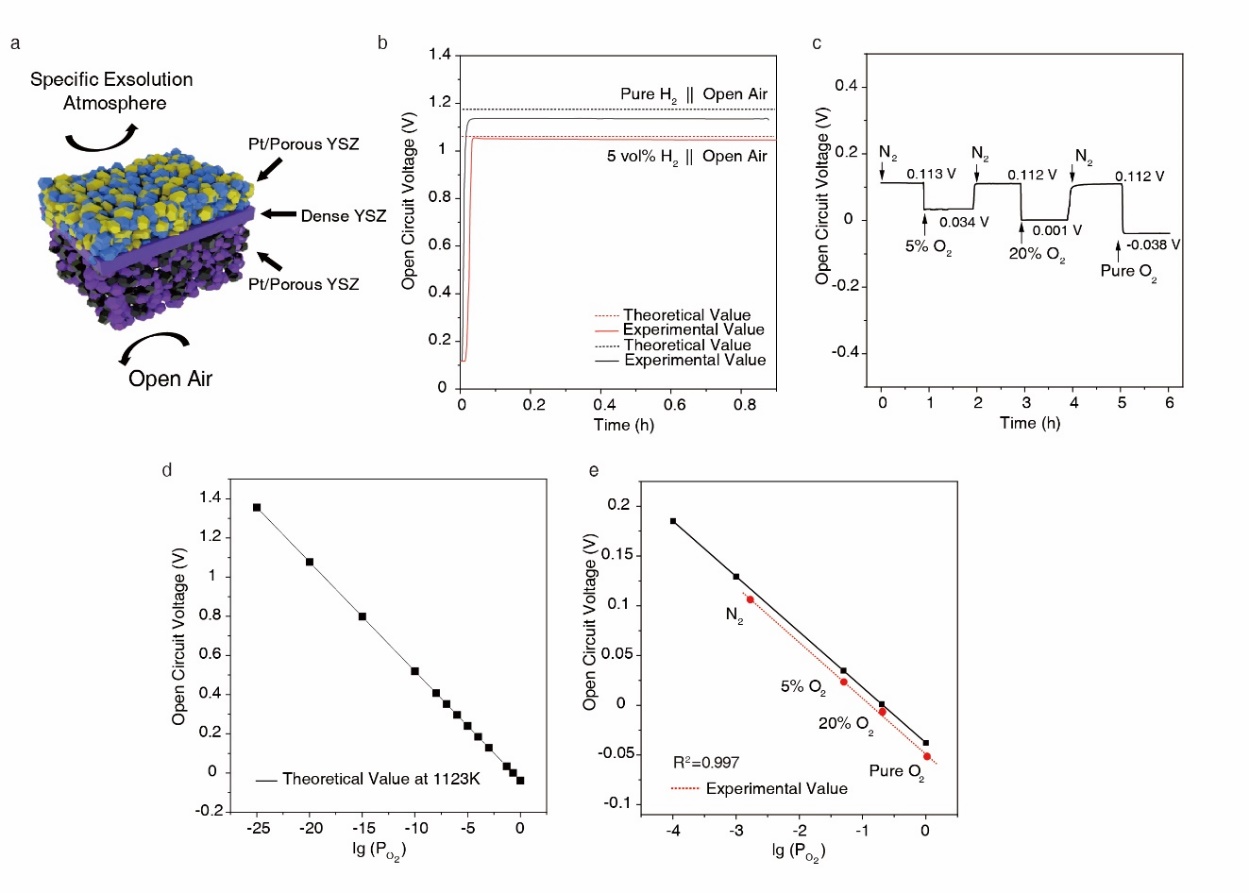


**FIGURE S5** Home-designed solid oxide cell for oxygen partial pressure (P_O2_) verification. a, The schematic illustration of home-made solid oxide concentration cell. The cell possesses a symmetric sandwich configuration of Pt (1 wt%) impregnated porous YSZ (anode, 20 μm) matrix / Dense commercial YSZ electrolyte (300 μm) / Pt (1 wt%) impregnated porous YSZ (cathode, 20 μm) matrix. The cathode was fed with ambient air with a flow rate of 50 mL min^-1^. b, The plot of experimental open circuit voltage (OCV) value at 1173 K. The cells with anode fed with pure H_2_ and 5% H_2_ fuels at the flow rate of 50 mL min^-1^ were measured and compared to theoretical OCV value calculated by Nernst Equation. The measured OCV value can reach 1.14 V and 1.04 V in H_2_ and 5% H_2_, respectively, which is very closed to the calculated values of 1.18 V and 1.06 V, indicating the reliability and accuracy of our cell. c, The on-line OCV value variation plot fed with different gases. While switching the gas to pure N_2_, a constant OCV of around 0.112~0.113 V can be obtained. And the 5% O_2_, 20% O_2_ and pure O_2_ can deliver an OCV of around 0.034 ,0.001 and -0.038 V, respectively. According to Nernst equation, the OCV of an electrochemical concentration cell can be expressed by the equation as follows (eq.1):

E=$\text{E}^{\text{0}}\text{+}\frac{\text{RT}}{\text{nF}}\ln\left( \frac{\text{P}_{\text{O2}}^{\text{cathode}}}{\text{P}_{\text{O2}}^{\text{anode}}} \right)\text{ }\text{ }\text{ }\text{ (}\text{eq.1)}$

Where $E^{0}$ is 0 V, R is gas constant of 8.314 J⋅K^−1^⋅mol^−1^, n is the electron transfer number, F is Faraday Constant (96500 C mol^-1^), T is the temperature. $P_{O2}^{cathode}$ and $P_{O2}^{anode}$ is the oxygen partial pressure (P_O2_) of the cathode and anode chambers of the cell, respectively. d-e, Calculated OCV value at 1123K at different P_O2_ values. The theoretical OCV value versus (P_O2_ in anode chamber) at 1123 K of our cell is shown in Figure S5d. And the comparison between theoretical value and experimental value is shown in Figure S5e. Obviously, the lg(P_O2_) and measured OCV has a good linear relationship with the R^2^ of 0.997. And the calculated P_O2_ of N_2_ can also be deducted to be around 10^-3^ Pa.


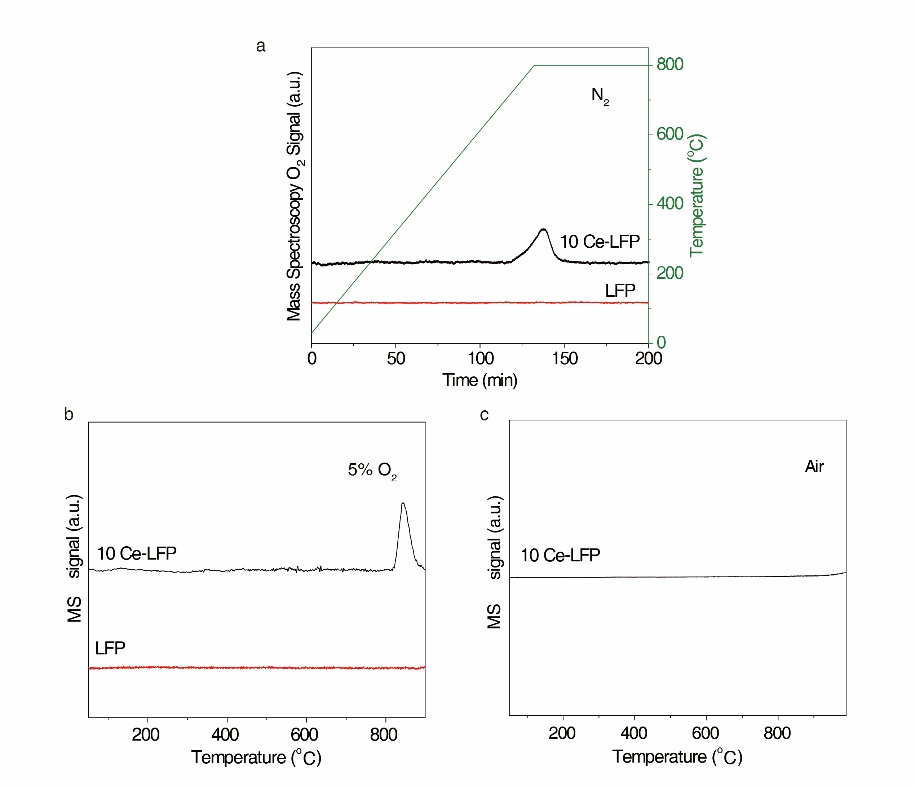


**FIGURE S6** The identification of signal for oxygen loss during *in-situ* exsolution using different atmospheres. a, The temperature dependent mass spectroscopy (MS) O_2_ signal (mass=32) in N_2_ atmosphere. The 10Ce-LFP was ramped at the rate of 10 ^o^C min^-1^ in N_2_ atmosphere from 30 ^o^C to 800 ^o^C (black line), and then isothermally kept at 800 ^o^C for another extra hour. Obviously, an O_2_ signal (mass=32) was detected while the temperature reached over 700 ^o^C, suggesting the release of O_2_ from perovskite oxide lattice in N_2_ atmosphere. This result indicates that the sample was destabilized and partially decomposed to form O_2_ and oxygen-deficient perovskite. And the destabilized structure will be balanced by the reduction of cation to metal: Pd^4+^ to Pd^0^. In comparison, the LFP sample without Ce (red line) displays no O_2_ (mass=32) peak, corresponding to no oxygen ion loss. b, MS O_2_ signal in 5% O_2_ atmosphere. The LFP catalyst (red line) shows no change of signal within the measurement temperature range. In comparison, the 10Ce-LFP sample (black line) shows a positive peak centering at 850 ^o^C, which is ascribed to the extra O_2_ release. This phenomenon is triggered by the difference of P_O2_ between perovskite lattice and atmosphere. c, MS O_2_ signal in air atmosphere of various samples. While switching the treatment gas and carrier gas to air, no O_2_ signal can be observed on 10Ce-LFP during the measurement range, indicating that the perovskite structure was maintained without weight loss and the exsolution process was not triggered.


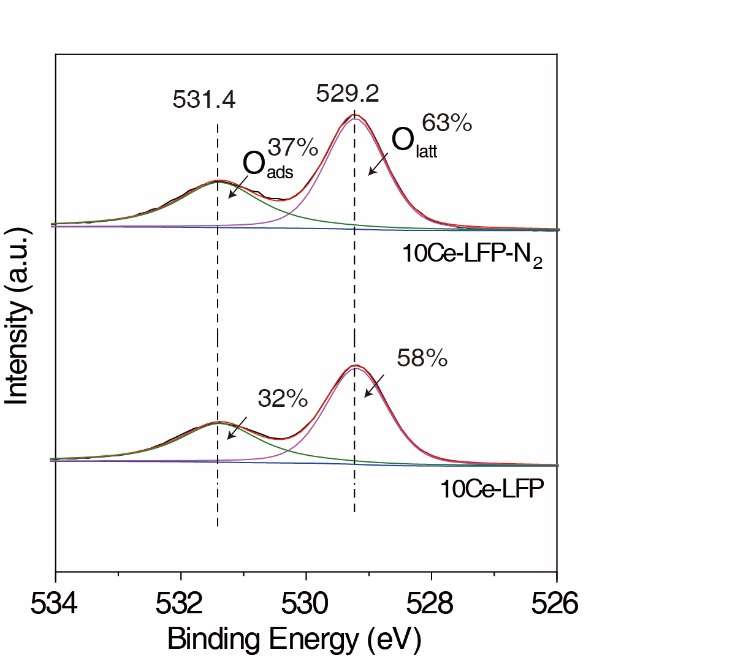


**FIGURE S7** The O1s XPS spectra of 10Ce-LFP sample before and after N_2_ treatment. The electronic configuration of surface oxygen species will change upon the N_2_ treatment, which can also greatly affect the reaction kinetics. XPS measurement was performed on10Ce-LFP catalysts before and after N_2_ treatment. The deconvoluted spectra were shown in Figure S7 as well. The O1s spectra presented two binding peaks centering at 529.2 eV and 531.4 eV, respectively. Generally, the peak at lower binding energy region (529.2 eV) is associated with the lattice oxygen species (O_latt_), whereas the one at higher binding energy region (531.4 eV) corresponds to the surface adsorbed oxygen species (O_ads_) loosely bonding on the surface, which is closely correlated with the surface oxygen vacancies.^[3]^ Apparently, 10Ce-LFP-N_2_ had higher proportions of surface oxygen vacancies (37%), as compared to its fresh counterpart (32%). The increase of surface defect concentration is tightly associated with the Pd segregation, which usually have higher energy states and might be important active sites for the O_2_ adsorption and activation.


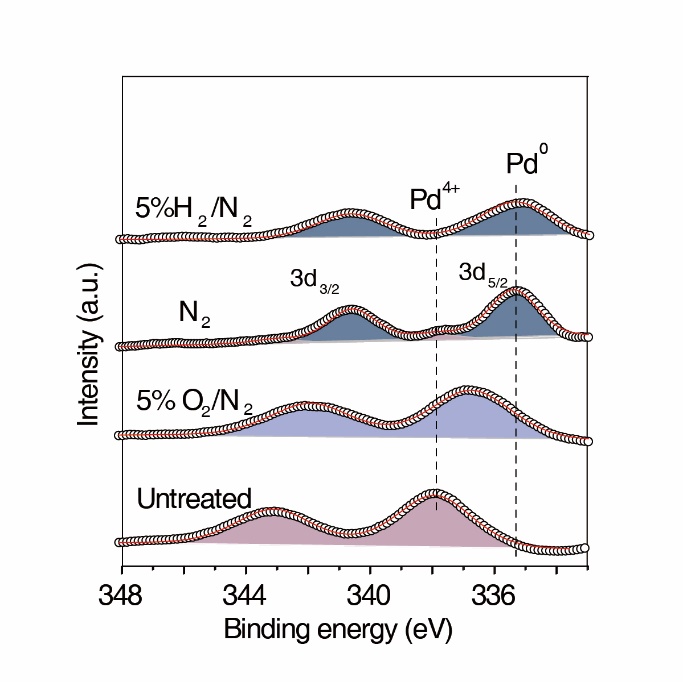


**FIGURE S8** Pd 3d XPS spectra of 10Ce-LFP treated by different atmospheres. The XPS analysis was further performed to investigate the surface chemical state of Pd element of catalysts treated with different atmospheres. For the freshly prepared catalyst, the single peak at 337.9 eV can be ascribed to Pd^4+^ 3d_5/2_ in PdO_6_ octahedral, which was similar to the previous report.^[4]^ After reduction in 5% H_2_/N_2_, the binding peak shift negatively to 335.3 eV, corresponding to Pd^0^ 3d_5/2_.^[5]^ It indicates that oxidative state of surface Pd exists entirely in metallic Pd after reduction. Similarly, the majority of Pd^4+^ (90%) was reduced to Pd^0^, while the sample was treated with N_2_ atmosphere. Interestingly, the 5%O_2_/N_2_ can also significantly manipulate the oxidative state of Pd^4+^ to Pd^2+^ with a single binding energy peak at 336.9 eV.^[6]^ This parallel comparison proves the possibility to precisely control electronic state of Pd by altering the treatment atmosphere with various P_O2_.


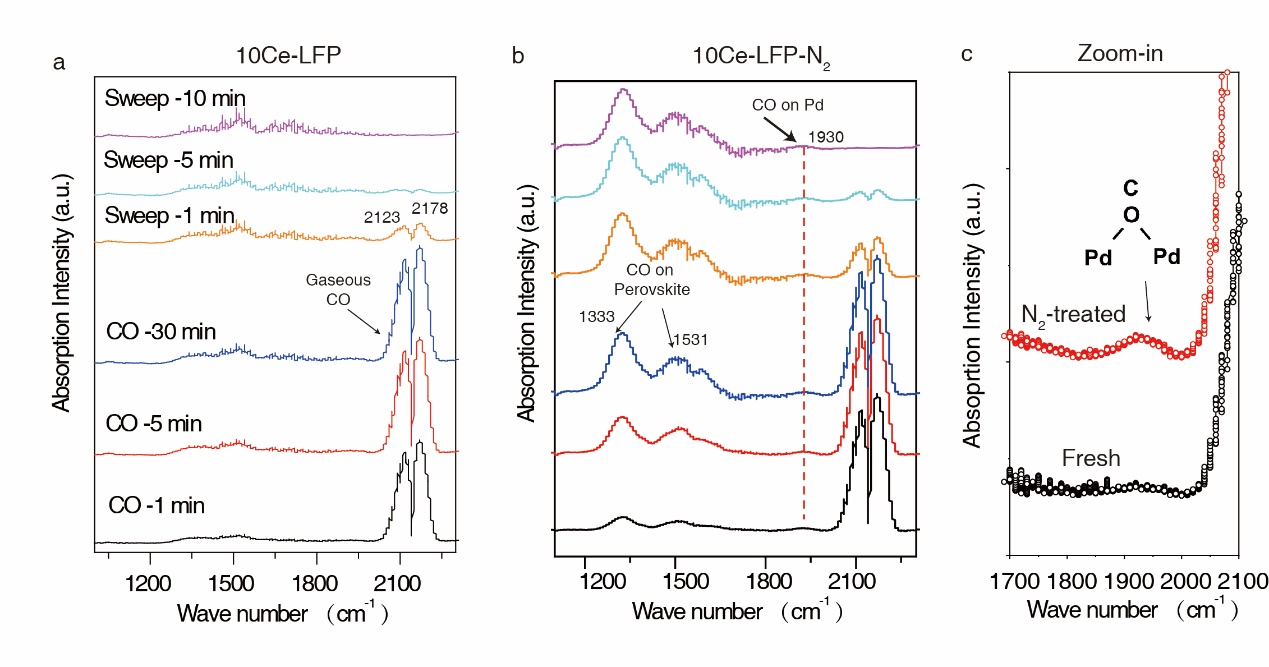


**FIGURE S9** CO DRIFT spectra. The CO DRIFT spectra of (a) fresh 10Ce-LFP and (b) 10Ce-LFP treated with N_2_. c, The zoom-in CO FT-IR plot of different samples treated with CO for 30 min. The peak at 2123 and 2178 cm^-1^ can be ascribed to the existence of gaseous CO, which disappeared while switching to sweeping gas of Ar for 15 min. And the peak at 1333 and 1511 cm^-1^ can be assigned to the CO absorption on perovskite oxide support.^[7]^ Interestingly, the intensity of the peak at 1930 cm^-1^, which is belonged to bridge absorption of CO on metallic state Pd, can only be seen on 10Ce-LFP after treating with N_2_.^[5]^ This result offers the supporting evidence for the transformation from Pd^4+^ to Pd^0^ by N_2_ treatment.


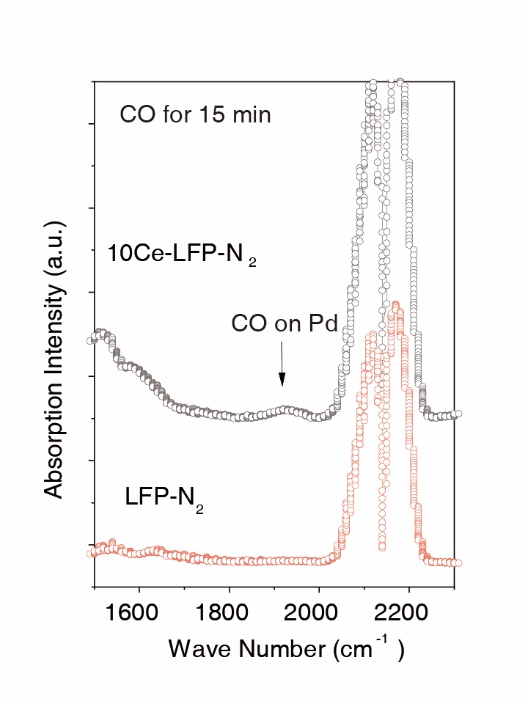


**FIGURE S10** The CO DRIFT spectra. The CO DRIFT spectra of 10Ce-LFP-N_2_ and LFP-N_2_ samples. The intensity of the bridge absorption of CO at 1930 cm^-1^ is much more obvious on 10Ce-LFP-N_2_ than that on LFP-N_2_, indicating that the doping of Ce can facilitate the formation of metallic Pd quantitively.

**Table S2** The atomic ratios of Pd/La+Ce for before and after N_2_ treated 10Ce-LFP catalysts.

| Sample | Binding energy (eV) | | | | Pd/La+Ce |
| --- | --- | --- | --- | --- | --- |
|  | Pd | | La | Ce |  |
| 10Ce-LFP | - | 338.2(Pd^4+^) | 833.8 | 897.3 | 3.7% |
| 10Ce-LFP-N2 | 335.3(Pd^0^) | - | 833.8 | 8983 | 8.5% |


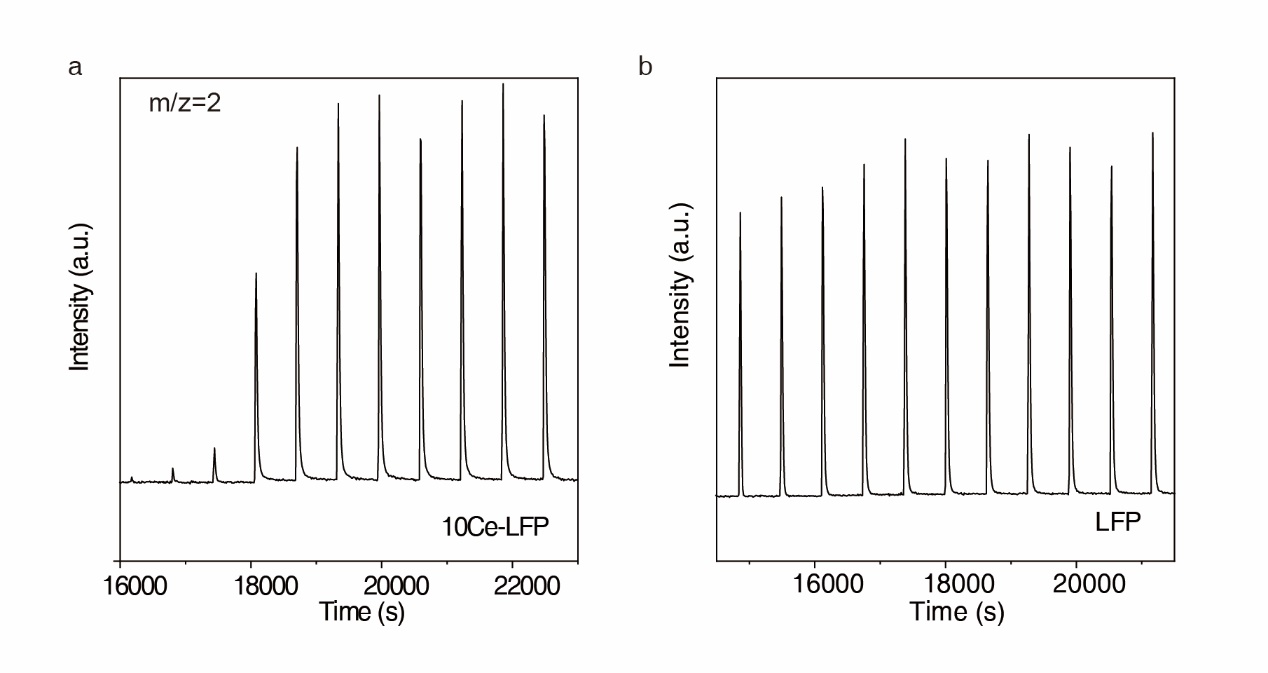

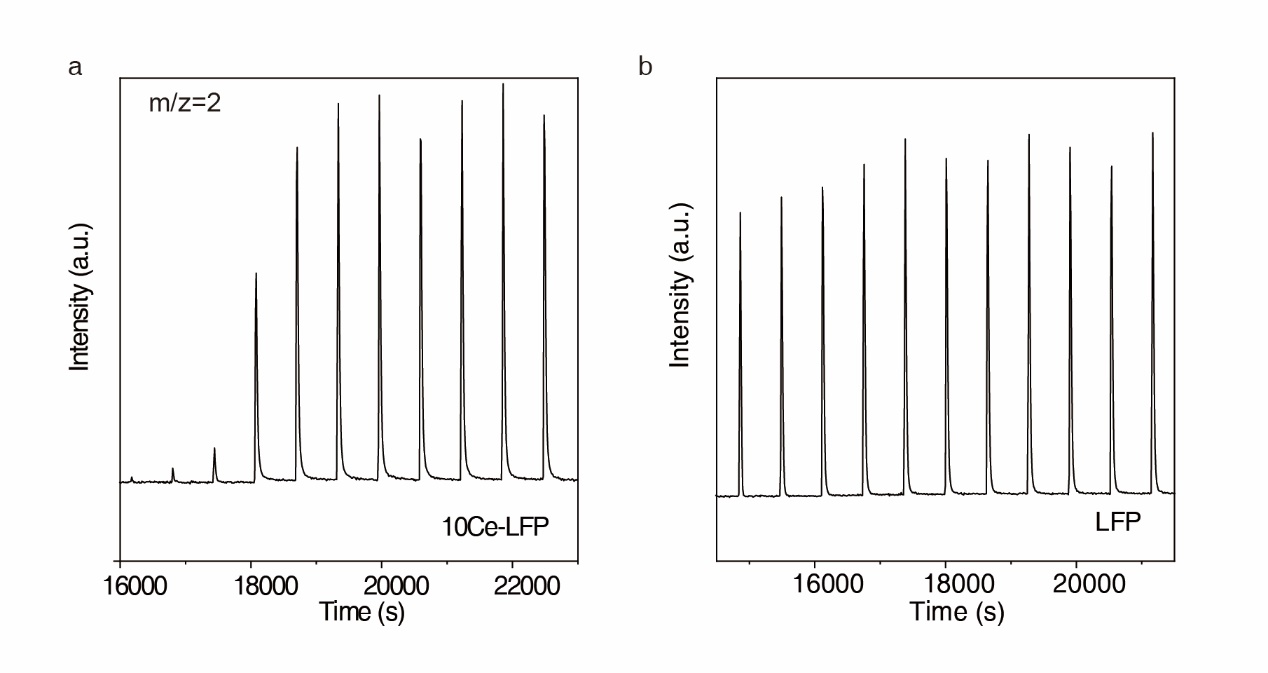


b

a

**FIGURE S11** The mass-spectrometry signal of H_2_ pulse experiment on (a) LFP and (b) 10Ce-LFP. After the reduction of catalysts by H_2_ and treatment of O_2_, the Pd cations were reduced to metallic Pd and the surface was saturated with O_2_ molecule. 1000μl of H_2_ was pulsed into the chamber, and the pulse was monitored by mass-spectrometry (m/z=2). The H_2_ was pulsed 11 times onto the reduced samples. Significant changes were observed up to the sixed pulse on 10Ce-LFP (a), while only weak change were detected up to fourth pulses on LFP. Such difference suggests the existence of Ce is directly correlated to the formation of larger amount of metallic Pd on 10Ce-LFP.


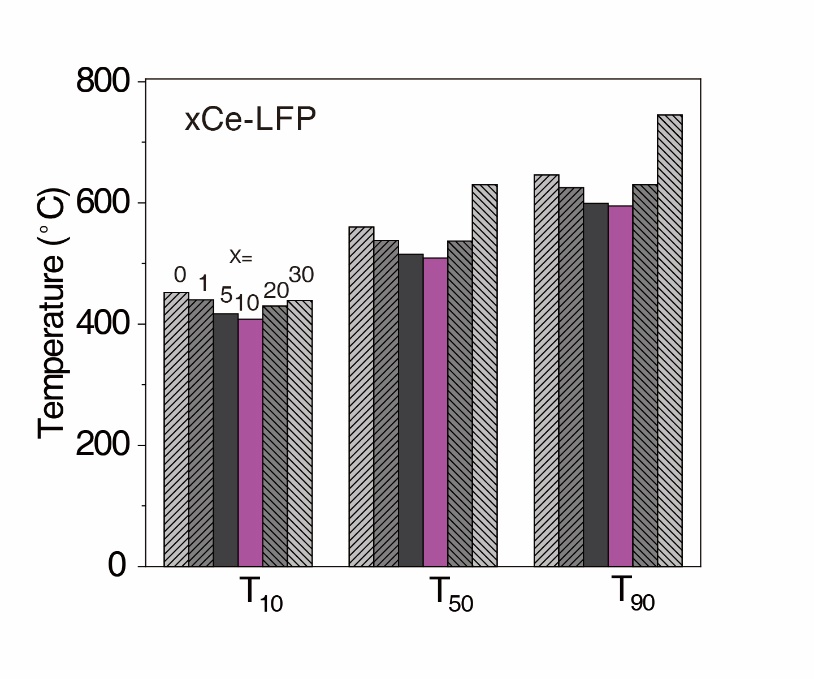


**FIGURE S12** The summary methane combustion performance over xCe-LFP catalysts (x = 0, 1, 5, 10, 20, 30). The plot of T_10_, T_50_ and T_90_ versus Ce concentration demonstrate a volcano-shape relationship and the 10Ce-LCP catalyst shows an outstanding T_10_ of 400 ^o^C, T_50_ of 510 ^o^C and T_90_ of 594 ^o^C, outperforming all other candidates. This result further stresses that 10 mol% is the optimal doping concentration.


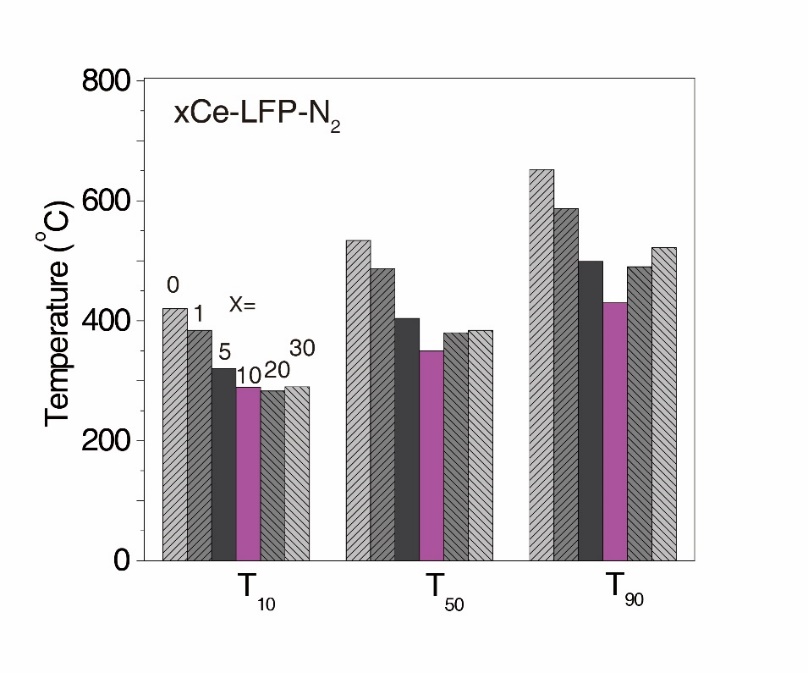


**FIGURE S13** The summary of methane combustion performance over xCe-LFP-N_2_ catalysts (x = 0, 1, 5, 10, 20, 30). The plot of T_10_, T_50_ and T_90_ versus Ce concentration also exhibits a volcano-shape relationship and the 10Ce-LCP-N_2_ sample show the best catalytic performance including T_10_ of 286 ^o^C, T_50_ of 350 ^o^C and T_90_ of 429 ^o^C.


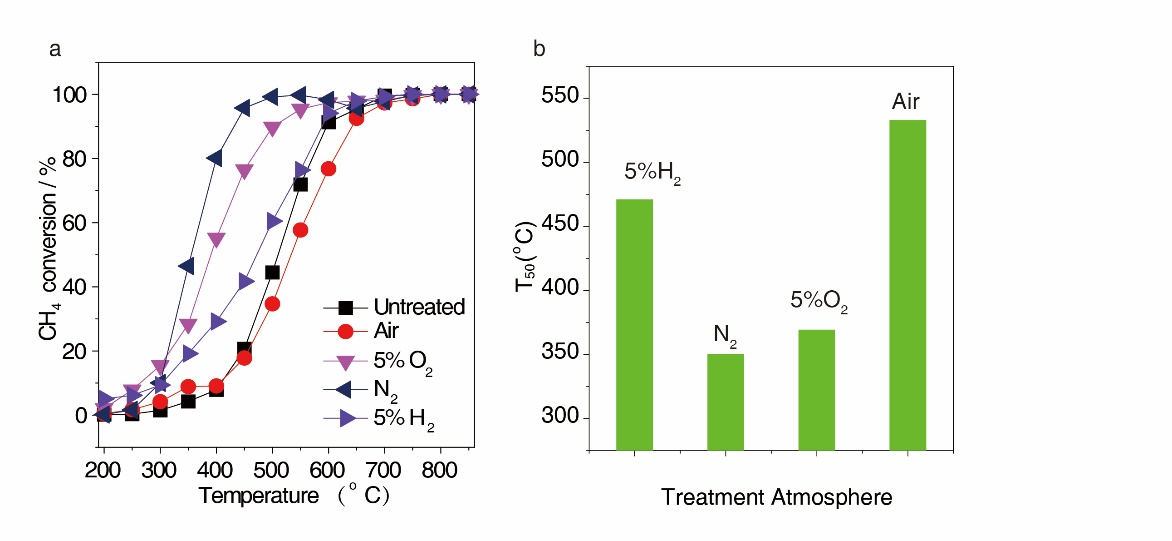


**FIGURE S14** Light-off curves for CH_4_ combustion over catalysts treated with different atmospheres. a, The Light-off curves. b, the comparison of T_50_ of various catalysts. The composition of treatment atmosphere significantly affects the modality of catalytic interface and further the activity. Different gas compositions lead to different P_O2_ which are related to the Pd species and oxygen vacancies. And both of them are reactive sites involved into the catalytic cycle of CH_4_ oxidation. Clearly, extremely reducing atmosphere (5% H_2_/N_2_ with low P_O2_) or oxidative atmosphere (pure air with high P_O2_) does not effectively promote the catalytic activity, and their T_50_ are both higher than 450 ^o^C. While the treatment of 5% O_2_/N_2_ obviously enhances the performance by reducing the T_50_ to 375 ^o^C which, however, is still higher than that of N_2_ treated samples (350 ^o^C).


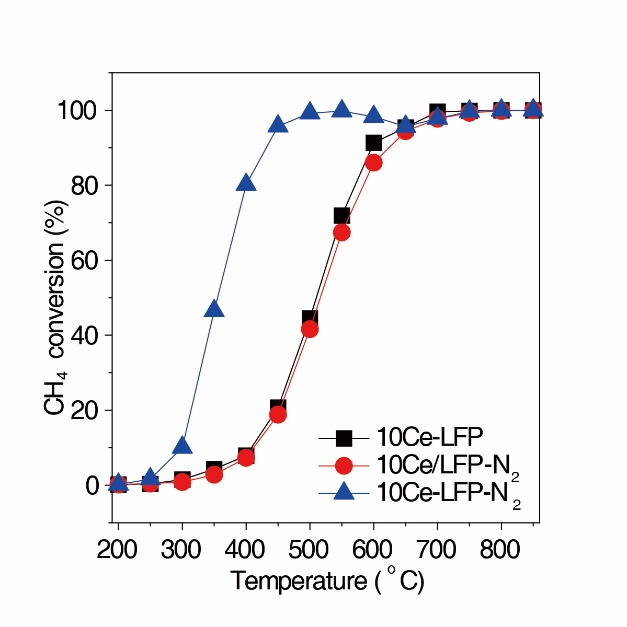


**FIGURE S15** Light-off curves for CH_4_ combustion over catalysts with various Ce states. Light-off curves for CH_4_ combustion in stoichiometric conditions over catalysts with different Ce states are also presented. In order to reveal the unique interaction between Ce in perovskite lattice and exsolved Pd cluster, the catalysts impregnated with 10 mol % Ce was also prepared, treated with N_2_ and employed to CH_4_ combustion reaction. The catalyst with infiltrated Ce demonstrates a poor methane combustion performance with the T_50_ of 530 ^o^C which is much higher than that of 10Ce-LFP-N_2_ (350 °C) and 10Ce-LFP (510 ^o^C). The discrepancy of catalytic performance can be explained by the intimate synergistic effect between highly dispersed Ce element and exsolved Pd cluster on 10Ce-LFP-N_2_. While the infiltrated Ce has poor distribution and weak interaction with Pd species.


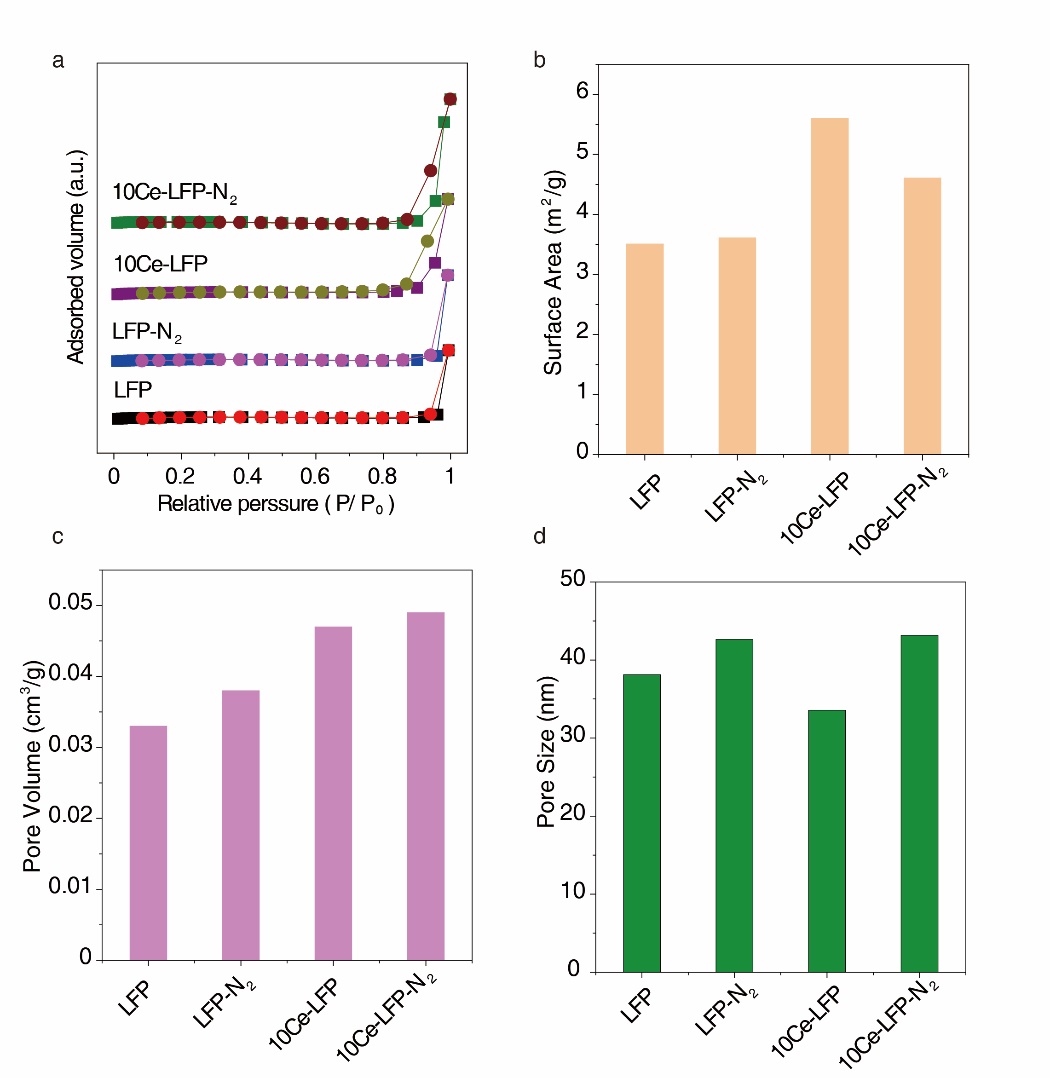


**FIGURE S16** Pore property of various catalysts. a, N_2_ adsorption–desorption isotherms curves. All catalysts show a type-IV isotherm with a hysteresis loop corresponding to typical mesoporous microstructure. The hysteresis ring for all catalysts is wide and the desorption curve is steeper than the adsorption curve, which usually appeared at porous materials with wide pore size. b-d, Summary of (b) BET surface area, c, pore volume and (d) pore size plot based on Barrett-Joyner-Halenda pore size distribution curves of various catalysts. All catalysts demonstrate a similar BET surface area ranging from 3.3 to 5.6 m^2^/g. And All samples demonstrate a quite identical pore properties, suggesting that the distinctive methane combustion performance lies in the difference of intrinsic activity of each reactive site.


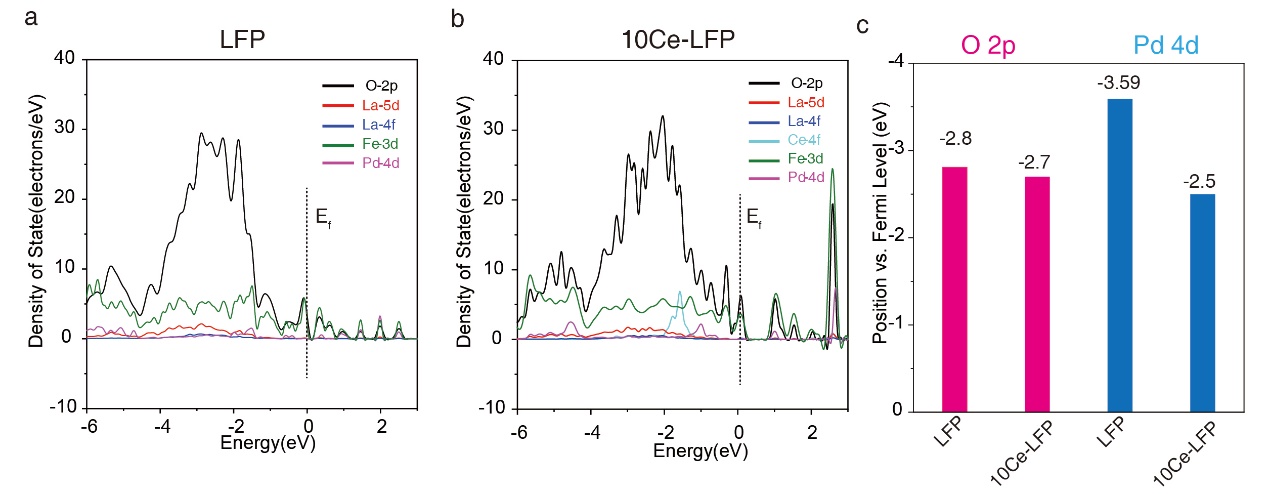


**FIGURE S17** Projected density of states (PDOS) plots of different catalysts. The PDOS plots of (a) LFP and (b) 10Ce-LFP are shown in Figure S16. The La-5d, O-2p, La-4f, Pd-4d, Fe-3d and Ce-4f states are shown in red, black, dark blue, purple, green and light blue colors, respectively. c, The summary of position of O 2p and Pd 4d versus. Fermi level. The integrated center position O 2p state is -2.81 eV and -2.78 eV for LFP and 10Ce-LFP catalysts, respectively. And the center position of Pd 4d is -3.59 eV and -2.50 eV, respectively. This calculation result provides direct evidence that the oxygen ion and Pd ion in 10Ce-LFP is more reactive and should be stripped off more easily.


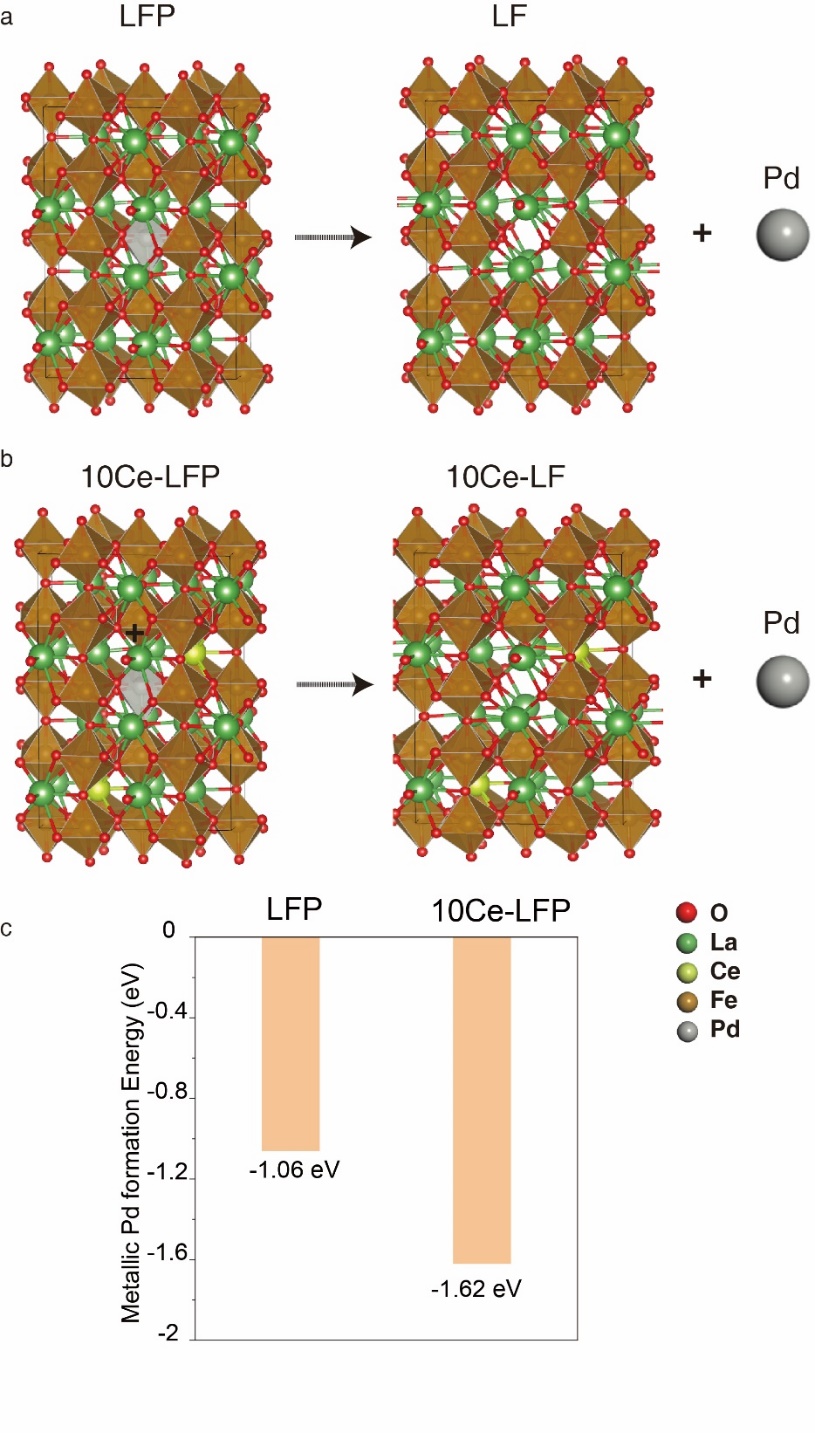


**FIGURE S18** Density Function Theory (DFT) calculations modes and metallic formation energy of 10Ce-LFP and LFP. a-b, The atomic structure of LFP (a) and 10Ce-LFP (b) model used for the calculations. The unit cell parameter of the materials and the 2×2×1 supercell (unit: Å) were also presented. c, The calculated metallic Pd formation energy of different perovskites. The formation energy of metallic Pd from LFP is -1.06 eV, which is much higher than that of 10Ce-LFP of -1.62 eV. This result suggests that the Ce additive tunes the reducibility of Pd and makes the exsolution of Pd more thermodynamically favorable.


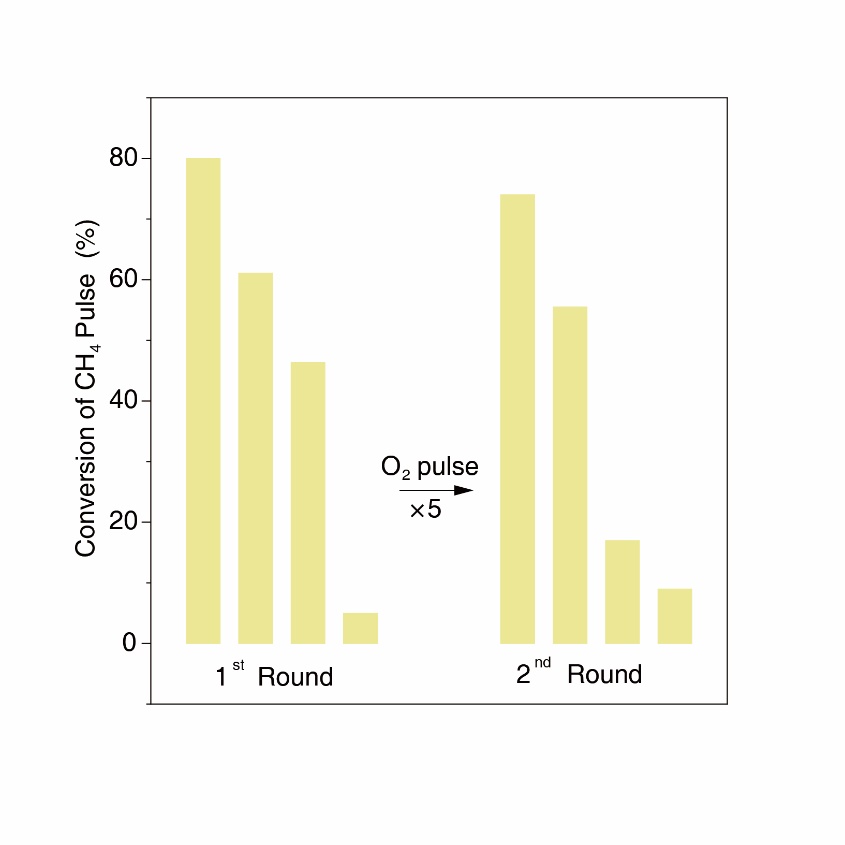


**FIGURE S19** Conversion of methane as a function of the pulse interval at 350 ^◦^C of 10Ce-LFP-N_2_ catalyst. The volume of quantitative loop is 1.197 mL. The 2%CH_4_/N_2_ was used. The methane conversion of the first pulse reaches 80% and decreases to around 4% after four consecutive pulses. This result implies that the lattice oxygen in 10Ce-LFP-N_2_ is active for CH_4_ conversion but consumed quickly. After 5 consecutive O_2_ pulse, the conversion of CH4 is recovered back to around 75%, suggesting that the refilling of oxygen vacancies by gaseous O_2_ is closely correlated to the recovery of CH_4_ conversion rate.


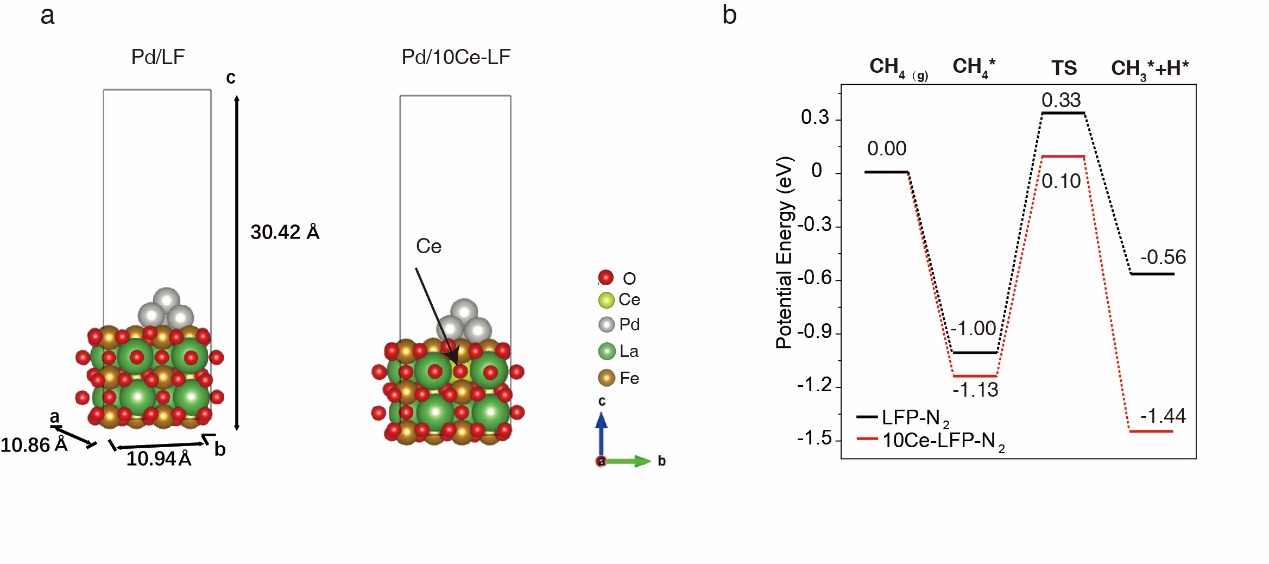


**FIGURE S20** The structure mode (a) and potential energies profiles (b). The CH_4_ activation pathway is simulated on (010) surface various catalysts. For10Ce-LFP-N_2_ surface, chemically adsorbed CH_4_ is present with a more negative adsorption energy of -1.13 eV, indicating CH_4_ molecule undergoes a strong adsorption and is easy to be dissociated. Further, it overcomes a lower energy barrier (-1.23 eV) to dissociate CH_4_ into one H and one CH_3_*.

**FIGURE S21** The fitted XPS spectra of the Ce 3d core for various catalysts. Due to the close proximity of the Ce 4f and O 2p orbitals, the Ce 3d XPS spectra is complex.^[8]^ In our catalyst, the binding energy positioning at 882.6 eV (v), 891.8 eV (v′), 898.9 eV (u), 907.3 eV(u″), and 916.4 eV (u″′) can be assigned to the Ce^4+^ chemical environment, and the peaks centering at 887.4 eV(v′) and 903.6 eV (u′) corresponds to the Ce^3+^.^[9]^ The fresh 10Ce-LFP catalyst obtained a Ce^3+^ concentration (Ce^3+^/(Ce^3+^+Ce^4+^)) of 20.2 %. After treatment of N_2_, the concentration of Ce^3+^ surges to 36.3 %, indicating the creation of more Ce^3+^/Ce^4+^ redox pairs.


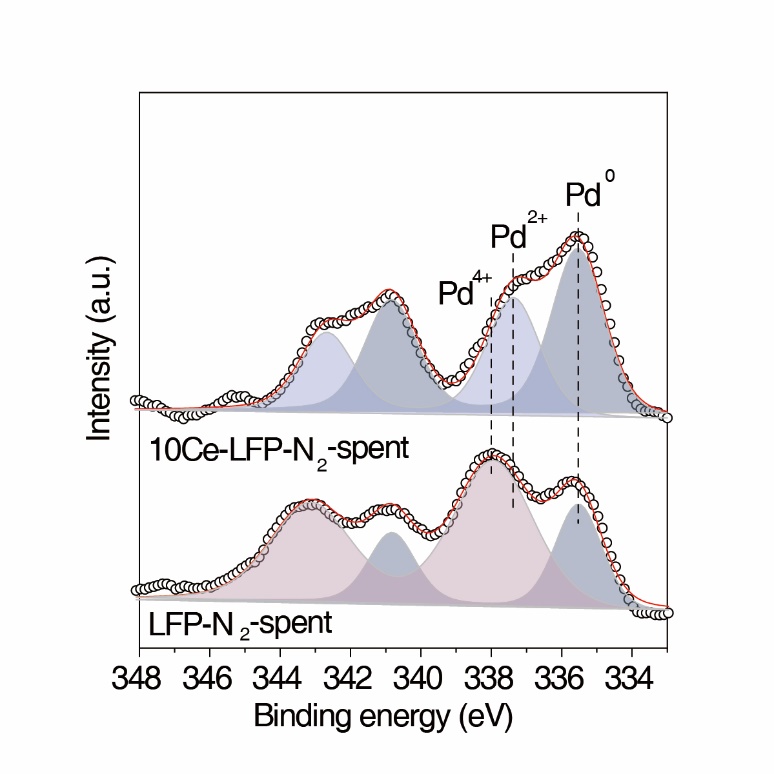


**FIGURE S22** The Pd 3d XPS spectra of samples after stability measurement. Initially, the pre-treatment of N_2_ leads to the surface exsolution of ultra-fine metallic Pd cluster. However, the reaction condition of methane combustion is in rich of O_2_ (CH_4_:O_2_:N_2_=1:6:93), which is expected to *in-situ* alters the surface electronic configuration of Pd species to form an intermediate state. Therefore, the catalysts after stability test (Figure 2e) was employed for XPS to figure out the real chemical valence information of Pd species. As shown in Figure S19, the binding peak ascribed to Pd^2+^ emerged at 337.4 eV with the co-existence of Pd^0^ peak at 335.5 eV on 10Ce-LFP-N_2_ catalyst after performance evaluation. The ratio of Pd^2+^/Pd^0^ is 0.42. In comparison, the spent LFP-N_2_ catalyst shows the co-existence of Pd^4+^ (69.9%) and Pd^0^ (30.1%) species with the XPS binding energy peaks centering at 338.2 and 335.4 eV, respectively. The comparison of XPS data elucidates that the prolonged measurement reduces the number of active sites by facilitating the formation of inactive Pd^4+^ species in the reaction conditions. And the additive of Ce can prevent the deep oxidation of Pd^0^ species and maintain a hybrid composition of Pd^2+^ and Pd^0^ which is active for methane oxidation.


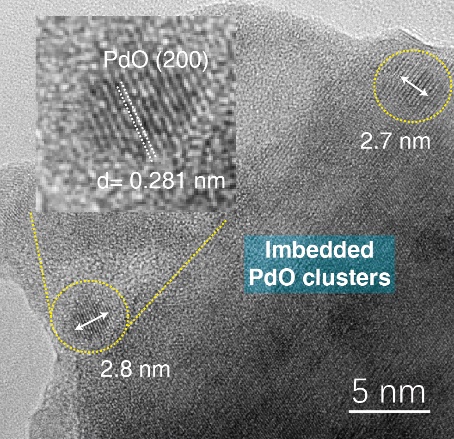


**FIGURE S23** The HR-TEM image of spent catalysts. The imbedded of PdO nanocluster with the diameter of around 2.8 nm can be observed in the HR-TEM image. The lattice fringe with distance of 0.281 nm can be ascribed to the (200) plane of PdO phase.

**REFERENCES**

1. B. Zheng, T. Gan, S. Shi, J. Wang, W. Zhang, X. Zhou, Y. Zou, W. Yan, G. Liu, *ACS Appl. Mater. Interfaces* **2021**, *13*, 27029.
2. R. Huang, C. Lim, M.G. Jang, J.Y. Hwang, J.W. Han, *J. Catal.* **2021**, *400*, 148.
3. B. Hua, Y.-Q. Zhang, N. Yan, M. Li, Y.-F. Sun, J. Chen, J. Li, J.-L. Luo, *Adv. Funct. Mater.* **2016**, *26*, 41062.
4. A. Eyssler, P. Mandaliev, A. Winkler, P. Hug, O. Safonova, R. Figi, A. Weidenkaff, D. Ferri, *J. Phys. Chem. C* **2010**, *114*, 4584.
5. J. Chen, J. Zhong, Y. Wu, W. Hu, P. Qu, X. Xiao, G. Zhang, X. Liu, Y. Jiao, L. Zhong, Y. Chen, *ACS Catal.* **2020**, *10*, 10339.
6. K. Murata, D. Kosuge, J. Ohyama, Y. Mahara, Y. Yamamoto, S. Arai, A. Satsuma, *ACS Catal.* **2019**, *10*, 1381.
7. L. Forni, C. Oliva, T. Barzetti, E. Selli, A.M. Ezerets, A.V. Vishniakov, *Appl. Catal. B* **1997**, 13, 35.
8. A. Kotani, T. Jo, J.J.A.i.P. Parlebas, *Adv. Phys.* **1988**, *37*, 37.
9. P.A. Connor, X. Yue, C.D. Savaniu, R. Price, G. Triantafyllou, M. Cassidy, G. Kerherve, D.J. Payne, R.C. Maher, L.F. Cohen, R.I. Tomov, B.A. Glowacki, R.V. Kumar, J.T.S. Irvine, *Adv. Energy Mater.* **2018**, *8*, 1800120.
